# SMYD1a protects the heart from ischemic injury by regulating OPA1-mediated cristae remodeling and supercomplex formation

**DOI:** 10.1007/s00395-023-00991-6

**Published:** 2023-05-22

**Authors:** Marta W. Szulik, Steven Valdez, Maureen Walsh, Kathryn Davis, Ryan Bia, Emilee Horiuchi, Sean O’Very, Anil K. Laxman, Linda Sandaklie-Nicolova, David R. Eberhardt, Jessica R. Durrant, Hanin Sheikh, Samuel Hickenlooper, Magnus Creed, Cameron Brady, Mickey Miller, Li Wang, June Garcia-Llana, Christopher Tracy, Stavros G. Drakos, Katsuhiko Funai, Dipayan Chaudhuri, Sihem Boudina, Sarah Franklin

**Affiliations:** 1 Nora Eccles Harrison Cardiovascular Research and Training Institute, University of Utah, Salt Lake City, UT USA; 2 Diabetes and Metabolism Research Center, University of Utah, Salt Lake City, UT USA; 3 Metabolic Phenotypic Core Facility, University of Utah, Salt Lake City, UT USA; 4 Electron Microscopy Core Laboratory, University of Utah, Salt Lake City, UT USA; 5 Dallas Tissue Research, Farmers Branch, TX USA; 6 Division of Cardiovascular Medicine, Department of Internal Medicine, School of Medicine, University of Utah, Salt Lake City, UT USA; 7 Department of Biochemistry, Department of Biomedical Engineering, University of Utah, Salt Lake City, UT USA; 8 Department of Nutrition and Integrative Physiology, Program in Molecular Medicine, University of Utah, Salt Lake City, UT USA

**Keywords:** SMYD1, Methyltransferase, Ischemic heart failure, Mitochondrial respiration, Cristae

## Abstract

SMYD1, a striated muscle-specific lysine methyltransferase, was originally shown to play a key role in embryonic cardiac development but more recently we demonstrated that loss of *Smyd1* in the murine adult heart leads to cardiac hypertrophy and failure. However, the effects of SMYD1 overexpression in the heart and its molecular function in the cardiomyocyte in response to ischemic stress are unknown. In this study, we show that inducible, cardiomyocyte-specific overexpression of SMYD1a in mice protects the heart from ischemic injury as seen by a > 50% reduction in infarct size and decreased myocyte cell death. We also demonstrate that attenuated pathological remodeling is a result of enhanced mitochondrial respiration efficiency, which is driven by increased mitochondrial cristae formation and stabilization of respiratory chain supercomplexes within the cristae. These morphological changes occur concomitant with increased OPA1 expression, a known driver of cristae morphology and supercomplex formation. Together, these analyses identify OPA1 as a novel downstream target of SMYD1a whereby cardiomyocytes upregulate energy efficiency to dynamically adapt to the energy demands of the cell. In addition, these findings highlight a new epigenetic mechanism by which SMYD1a regulates mitochondrial energetics and functions to protect the heart from ischemic injury.

**Supplementary Information:**

The online version contains supplementary material available at 10.1007/s00395-023-00991-6.

## Introduction

Ischemic heart disease is the primary cause of chronic heart failure (HF), one of the most devastating conditions with mortality exceeding 50% in patients diagnosed with advanced HF [60]. Although significant advancements have been made clinically to reduce myocardial injury from ischemia and subsequent reperfusion (including stents and bypass surgery), currently 25% of patients will die or develop heart failure within one year [13]. Therefore, understanding the molecular pathways that contribute to or protect from ischemic injury may be useful for developing novel therapies to prevent or slow the progression of heart failure or reverse the functional deficiencies of the failing heart [25, 58].

There have been many studies addressing molecular mechanisms underlying ischemic injury that focus on myocardial metabolism and energetics however, we know very little about the epigenetic regulators, which induce transcriptional changes in the myocyte genome and drive these metabolic changes [9, 33, 47]. While we have made advancements in the field of epigenetics in general, the proteins responsible for regulating chromatin and their subsequent effects on cellular and organ remodeling during ischemic heart disease are largely unknown. Studies of acute myocardial infarction in animal models have shown that targeting key pathways in the cardiomyocyte can diminish ischemic injury [49, 66]. Specifically, early reports utilizing histone deacetylase inhibitors revealed that heart disease progression could be attenuated upon treatment in animal models; however, the ubiquitous expression of these proteins and the non-specific nature of the inhibitors has made them unfeasible as a therapeutic tool in the heart, thus far [6]. In contrast, the lysine methyltransferase SMYD1 is a myocyte-specific epigenetic regulator, which has been shown to regulate gene expression in the cardiomyocyte. Originally, SMYD1 was reported to play a role in embryonic cardiac development, however, more recently SMYD1 has been shown to be differentially expressed in human heart failure patients and in mouse models of heart disease [7, 20, 23, 42]. Our previous work focused on the characterization of *Smyd1*-knockout mice and demonstrated that cardiomyocyte-specific loss of *Smyd1* in the adult murine heart leads to myocyte growth and heart failure, which is preceded by dysregulation of cardiac metabolism and a reduction in mitochondrial respiration capacity [20, 62]. In addition, we showed in cultured cells that SMYD1’s ability to regulate mitochondrial respiration was due, in part, to its transcriptional control of *Ppargc1α* (a regulator of mitochondrial number and energetics) [20, 62]. However, the physiological ramifications of SMYD1 gain-of-function in an animal model as well as its role in response to ischemic injury have never been elucidated.

Here we describe a newly generated mouse model capable of inducible, cardiomyocyte-specific overexpression of SMYD1a in transgenic mice (TG) and demonstrate for the first time that SMYD1a gain-of-function can positively regulate mitochondrial respiration in an animal model. This enhanced respiration protects the heart from ischemic injury and reduces infarct size by > 50%. These mice are also capable of maintaining basal levels of PGC-1α expression and its downstream targets, including electron transport chain subunits, after ischemic injury. In addition, our data from SMYD1a overexpressing mice show increased mitochondrial cristae formation and stabilization of respiratory chain supercomplexes within the cristae, concomitant with increased OPA1 expression, a known driver of cristae morphology. Together these results demonstrate that SMYD1a protects from ischemic injury by regulating mitochondrial energetics and enhancing respiration efficiency in the cardiomyocyte through the synergistic regulation of both PGC-1α and OPA1. This work highlights that SMYD1a is the only known epigenetic regulator of cristae morphology. It provides broad implications for understanding the epigenetic mechanisms driving cardiac energetics and identifies a novel signaling pathway by which cardiomyocytes regulate energy efficiency, protecting them from ischemic injury.

## Materials and methods

### Generation of transgenic mice which overexpress Smyd1a

Experiments were carried out according to the NIH Guidelines on the use of Laboratory Animals, and all the protocols and procedures were approved by the Institutional Animal Care and Use Committee at the University of Utah. To achieve cardiomyocyte-specific overexpression of mouse ortholog of human SMYD1, which is histone lysine N-methyltransferase Smyd1 isoform 1 (GenBank: NM_001160127.1), referred to as *Smyd1a*, animals expressing a reverse tetracycline-controlled transactivator (*rtTA*) under the *αMHC* promoter (acquired from Drs. Dale Abel and Adam Wende [63]) were crossed with mice carrying the *Smyd1a* gene (tagged with FLAG on the C-terminus) under a modified *αMHC* promoter containing a TRE (7 tet-o repeats) element, which was developed in the University of Utah’s Transgenic and Gene Targeting Core. Resulting animals were *Smyd1a*^TRE+/−^/*rtTA*^+/−^, referred to as transgenic (TG) or SMYD1a-OE, and *Smyd1a*^TRE−/−^/*rtTA*^+/−^ referred to as WT littermate controls. Animals generated were maintained on an FVB background, housed under standard conditions, and given food and water ad libitum. Primers used for genotyping are listed in Supplemental Table S3.

To induce SMYD1a overexpression, mice were fed a special chow diet containing 625 mg/kg of doxycycline hyclate (TD.130141 from Envigo) at a daily dose of 2–3 mg for the length of the study. DOX-induced expression of SMYD1a in the heart was confirmed by qPCR, western blotting and LC–MS/MS in at least three separate experiments.

### Smyd1-knockout mice

Inducible, cardiomyocyte-specific *Smyd1*-knockout mice (*Smyd1*^flox/flox^ Cre^+/−^) and control mice (*Smyd1*^flox/flox^ Cre^−/−^) were developed as previously described [20, 62]. Mice at 8 weeks of age were fed tamoxifen containing chow (0.4 mg/g of chow diet, TD.07262, Harlan) for 3 weeks at which time their hearts were harvested, ventricles separated and immediately frozen in liquid nitrogen and stored at − 80 °C until they were used for analysis.

### Surgeries

Permanent occlusion (PO) of the left anterior descending artery (LAD) or Sham surgeries were performed 2 weeks after DOX-induced overexpression of SMYD1a in adult (16–19 week) SMYD1a-transgenic (TG) mice and their littermate controls (WT), with continued doxycycline chow administration. Surgeries were performed using aseptic techniques in a dedicated surgery facility. Animals were anesthetized with 2% isoflurane. In the supine position, endotracheal intubation was performed, and mice were ventilated with a small animal respirator. After the pericardiectomy, the left anterior descending (LAD) coronary artery was visualized under the microscope. A suture was placed through the myocardium underneath and around the LAD, approximately midway between the apex and the base, and tied with a surgeon’s knot. The apex of the left ventricle was observed for evidence of myocardial blanching indicating interruption in coronary flow. The Sham (control) procedure was identical except that the LAD was not ligated. By ligating the LAD, it is expected that the majority of blood flow through this artery is blocked, however, collateral circulation in the heart will continue to provide some blood flow to tissue in the region. The mice were deidentified (regarding genotype) and randomly assigned to a surgeon who was blinded to the genotypes during the surgical procedures to remove any possible bias. Cardiac function was monitored prior to and once a week following PO surgeries as detailed below by echocardiography. Mice were ultimately euthanized at 24 h, 48 h, or 5 weeks post-operatively to obtain cardiac tissue for histological and biochemical analyses.

### Mouse cardiac tissue collection

Cardiac tissue was harvested from mice and rinsed with phosphate-buffered saline (PBS), drained and weighed, followed by atrial and vascular tissue removal to leave the ventricles. Heart weight (HW), body weight (BW) and tibia length (TL) were measured to calculate HW/BW and HW/TL ratios for evaluation of hypertrophic response to PO. Extracted heart ventricles were cut into four 1.5 mm sections using a mouse heart slicer matrix (Zivic Instruments). Tissue sections, except for histology, were immediately frozen in liquid nitrogen and stored at -80 °C until use.

### Human cardiac tissue

We prospectively enrolled patients (age ≥ 18 years) in institutions comprising the Utah Transplantation Affiliated Hospitals (U.T.A.H.) Cardiac Transplant Program (i.e. University of Utah Health Science Center, Intermountain Medical Center, and the Veterans Administration Salt Lake City Health Care System) with clinical characteristics consistent with dilated cardiomyopathy and chronic advanced heart failure who required circulatory support with continuous flow Left Ventricular Assist Device (LVAD) as a bridge to transplantation or lifetime destination therapy. Patients who required LVAD support due to acute heart failure (acute myocardial infarction, acute myocarditis, post-cardiotomy cardiogenic shock, etc.) were prospectively excluded. LVAD patients underwent serial echocardiograms monthly for the first three months and at 4.5 and 6 months. They were categorized as either responders or non-responders using left ventricular ejection fraction (LVEF) and left ventricular end-diastolic diameter (LVEDD) measurements during diminished LVAD support “turn-down” echocardiograms). Responders were defined as patients with an LVEF > 40% and LVEDD ≤ 5.9 cm at six months post LVAD implantation, whereas non-responders were defined as patients with an LVEF < 35% and with < 50% relative improvement in LVEF regardless of the final LVEDD. The study was approved by the institutional review board of the participating institutions, and informed consent was provided by all patients. For heart failure patients, their clinical demographics, echocardiographic parameters, protein biomarkers and other clinical data were prospectively collected and entered into our program’s research electronic data capture system (REDCap). Myocardial tissue from donor’s hearts, not allocated for heart transplantation due to non-cardiac reasons (size, infection, etc.), was used as non-failing control. Myocardial tissue was prospectively collected from the LV apical core at the time of LVAD implantation and was snap frozen before storing it at − 80 °C, as described before [15, 16]. Donor control samples were acquired from hearts that were not transplanted due to non-cardiac reasons and LV apical tissue was harvested and processed the same way as the failing hearts. Clinical characteristics of the study population from subjects with advanced heart failure grouped as responders and non-responders as well as from donors’ group is presented in Supplementary Table S1 and S2, respectively.

### Echocardiography

In vivo cardiac function of mice was performed by echocardiography, as described previously [19, 20], using a Vevo 2100 ultrasound machine in the Small Animal Ultrasound Core at the University of Utah. The following indices were examined: left ventricular size (end diastolic and systolic dimensions), wall thickness, ventricular mass, and ventricular function (ejection fraction and fractional shortening).

### Histological analysis

Heart tissue from transgenic (TG) mice and their littermate controls (WT) were harvested for histology analyses and evaluation for gross morphology and cellular level parameters, as previously described [20]. Briefly, hearts were fixed using 4% paraformaldehyde and submitted to the Biorepository and Molecular Pathology Shared Resource at Huntsman Cancer Institute at the University of Utah, for paraffin embedding and sectioning (4 μm). Tissue sections were stained with Masson’s trichrome and Hematoxylin and Eosin (H&E). Sections were visualized using an Olympus BX51WI microscope with a DP73 color camera and image analysis was performed with CellSens software (Olympus). To quantify the area of infarct: trichrome and H&E slides were evaluated by an ACVP-board certified veterinary pathologist. One MT image per heart (*n* = 5–9/group was imported to ImageJ software for measurement of midline infarct length. The infarct size was calculated as infarct length/left ventricular circumference*100. Samples with lesions affecting < 50% of the thickness of the ventricle were recorded as “0” for the infarct size [55]. H&E-stained slides (*n* = 3–4/group) containing multiple sections of the heart per animal were also scored according to the extent of lesioned area: 0 = absent, 1 = minimal (< 10% affected), 2 = mild (10–25% affected), 3 = moderate (26–50% affected), 4 = marked (51–75% affected), 5 = severe (> 75% affected) [22]. The scores for each section were summed for individual animals.

### High-resolution respirometry

To induce cardiac-specific overexpression of SMYD1a, the transgenic mice were maintained on doxycycline chow for 2 weeks before harvest. Mitochondrial O_2_ utilization was measured using the Oroboros O2K Oxygraphs, as previously described [17, 29, 37]. Cardiac tissue was minced in a mitochondrial isolation medium (300 mM sucrose, 10 mM HEPES, 1 mM EGTA at pH 7.2) and subsequently homogenized using a Teflon-glass system. Homogenates were then centrifuged at 800 × g, 4 °C for 10 min, after which the supernatant was separated and centrifuged at 12,000 × g, 4 °C for 10 min. The resulting pellet was carefully resuspended in a mitochondrial isolation medium. Isolated mitochondria in the amount of 12.5 mg/mL were then added to the Oxygraph chambers containing assay buffers (105 mM MES potassium salt, 30 mM KCl, 10 mM KH_2_PO_4_, 5 mM MgCl_2_, 0.5 mg/mL BSA). Complex I/II supported respiration and maximal respiration was measured in response to the following substrates added in saturating concentrations in sequence: 0.5 mM malate, 5 mM pyruvate, 2.5 mM ADP, 5 mM glutamate, 10 mM succinate, 1.5 mM FCCP. Data were processed using Oroboros DatLab7 software and statistical analysis was performed using Excel.

### ATP production

Mitochondria were isolated as described above. To quantify ATP production per O_2_ reduction (ATP:O ratio) we used high-resolution respirometry (Oroboros O2k oxygraphs) as described above, coupled with fluorometry (Horiba Fluoromax-4), as previously described [35]. Briefly, isolated mitochondria in the amount of 2.5 mg/mL were added to the fluorometer chamber containing assay buffers (105 mM MES potassium salt, 30 mM KCl, 10 mM KH_2_PO_4_, 5 mM MgCl_2_, 0.5 mg/mL BSA, 1 U/mL Hexokinase, 2.5 U/mL Glucose-6-Phosphate Dehydrogenase, 2.5 mM D-glucose, 2.5 mM NADP + , 200 μM P1, P5-di(adenosine-5′) pentaphosphate). ATP production was measured in response to the following substrates: 0.5 mM malate, 5 mM pyruvate, and 2.5 mM ADP. Data were processed using Oroboros DatLab7 software and Excel and statistical analysis was performed using Excel.

### Cell mito stress test

To evaluate the mitochondrial function, the cell mito stress test was performed in H9c2 cells using a Seahorse XFe96 Flux Analyzer (Agilent), as previously described [62]. Briefly, the H9c2 cells were plated in 96-well Seahorse analyzer plates pre-coated with laminin (20 k cells per well) and transduced with adenovirus carrying SMYD1a-FLAG at a multiplicity of infection (MOI) of 100. Cells not treated with adenovirus were used as controls. After 48 h, oxygen consumption rates (OCR) were measured in an XF base medium (Agilent-Seahorse) at pH 7.4 and supplemented with 2 mM L-glutamine, 1 mM pyruvate and 25 mM glucose. The OCR rates were measured before and after the sequential addition of inhibitors: oligomycin (1 μM), carbonyl cyanide 4-(trifluoromethoxy) phenylhydrazone (FCCP, 5 μM) and a mix of rotenone (1 μM) and antimycin A (1 μM). The OCR values were normalized to the intensity of nuclear staining. For the palmitate oxidation assay, 24 h hours after adenovirus transductions, H9c2 cells were incubated in substrate-limited media containing DMEM formulated without glucose, glutamine, sodium pyruvate and HEPES, and supplemented with 0.5 mM glucose, 1 mM GlutaMAX, 0.5 mM l-carnitine, and 1% FBS for 24 h prior to the assay, followed by incubation with either palmitate (160 μM) coupled to BSA or BSA alone in Krebs-Hanseleit buffer at pH 7.4 containing 5 mM L-carnitine and 5 mM HEPES. The OCR rates in response to inhibitors were measured as described above.

### TUNEL assay

TUNEL assay was performed on cardiac tissue or cultured H9c2 cardiomyoblasts using In Situ Cell Death Detection Kit, Fluorescein, Sigma Aldrich according to the manufacturer’s instructions. Briefly, paraffin-embedded tissues were cut into 5 μm-thick sections, dewaxed, rehydrated according to standard protocols. H9c2 cardiomyoblasts were plated on laminin-treated coverslips and after 24 h transduced with Ad-SMYD1a or empty virus (control). After 48 h cells were subjected to hypoxia after which they were fixed and permeabilized according to standard protocols. Samples were treated with Proteinase K, then labeled using a TUNEL reaction mix. Tissue sections were stained for cardiomyocytes using standard immunohistochemistry methods with cardiac troponin C antibody (Abcam, ab137130). Then samples were washed with 1X PBS before mounting on coverslips using an aqueous-based fluorescence mounting medium containing DAPI (Prolong Gold). Negative controls were used to exclude false positives by incubating fixed and permeabilized sections in label solution without terminal transferase instead of the TUNEL reaction mixture. Positive controls were generated by incubating fixed and permeabilized sections with recombinant DNase I to induce DNA strand breaks. Images were acquired using a fluorescence microscope with an excitation wavelength in the range of 450–500 nm and detection in the range of 515-565 nm (green). The TUNEL-positive rate was calculated and expressed as the number of TUNEL-positive cells divided by the total number of nuclei counted.

### Isolated neonatal rat cardiomyocytes

Neonatal Rat Ventricular Myocytes (NRVMs) were isolated from Sprague–Dawley neonatal rats using the Neonatal Cardiomyocyte Isolation System kit (Worthington), as previously described [20, 62]. Briefly, NRVMs were isolated by enzymatic digestion from 0–1 day old litters and plated at 50% confluency in growth medium (DMEM supplemented with 10% FBS media, 1% penicillin–streptomycin (P/S), l-glutamine, d-glucose) for 24 h and maintained in serum-free media thereafter (DMEM supplemented with 1% insulin-transferrin-selenium (ITS), 1% P/S, l-glutamine, d-glucose). Overexpression of SMYD1a was induced using an adenovirus carrying FLAG-tagged SMYD1a or an empty virus as control at an MOI of 25.

### Cultured H9c2 cardiomyoblasts and imaging

H9c2 cardiomyoblasts were cultured at 70% confluency in a growth medium (DMEM supplemented with 10% FBS, 1% penicillin–streptomycin (P/S). *Smyd1* or *Opa1* knockdown was induced using respective siRNAs (Qiagen) or scrambled-siRNA (Qiagen) as a control, and lipofectamine RNAiMAX reagent (Thermo Fisher Scientific) in OPTI-MEM (Gibco). For the overexpression study, H9c2 cardiomyoblasts were transduced with adenovirus expressing SMYD1a-FLAG (Ad-SMDYD1a) or empty virus (Control) at a multiplicity of infection (MOI) of 150. All cells were cultured for 48 h or 72 h before harvest for end-point experiments. For ROS and calcium imaging, cells were cultured in normoxia for 48 h after transduction with adenovirus, followed by 4 h incubation in hypoxic conditions, as described below. Approximately 30 min before the end of 4 h-incubation time cells were loaded with 5 μM MitoSox (Thermo Fisher Scientific), or 5 μM X-Rhod1 (Thermo Fisher Scientific) and returned to the hypoxic chamber for the remainder of the time. Immediately after this time cells were imaged with a Zeiss LSM 510 confocal microscope. For either dye, measurements were performed with excitation and emission at 548 nm and 574 nm, respectively. All cells were imaged at the same power and gain settings. Image analysis was performed using Cell Profiler [41] as previously published [17].

### Hypoxia protocol for cultured cells

Cells were cultured in hypoxic conditions, as previously published [17]. Briefly, a glucose-free, serum-free “hypoxic” media was made using Dulbecco's modified eagle's medium (DMEM) without glucose, l-glutamine, phenol red, sodium pyruvate or sodium bicarbonate (Sigma, D5030). Potassium concentration of the media was doubled by adding 0.4 g/L KCl and the pH was adjusted to 6.5 to simulate typical ischemic conditions of hyperkalemia and acidosis. The “hypoxic” DMEM was gassed with 95% nitrogen, 5% carbon dioxide for 1 h prior to use to expel any excess oxygen. H9c2 cardiomyoblasts were placed in a sealed container at 37 °C with a constant gas flow of 95% nitrogen, 5% carbon dioxide to ensure complete depletion of oxygen from the solution. Hypoxia lasted for 4 h, after which cells were removed from the container and immediately imaged. Control cells were kept at 37 °C for the same amount of time that the experimental cells were exposed to hypoxic conditions. Imaging took place immediately following hypoxia.

### Calcium retention capacity (CRC)

The CRC assay was performed as previously published [17, 52]. Briefly, imaging was performed in a 96-well plate on a Cytation 5 microplate reader. Cells (0.5 million) were incubated in 100 μL of imaging solution containing (mM): 125 KCl, 20 HEPES, 5 K_2_HPO_4_, 1 MgCl_2_, and 10 μM EGTA (pH to 7.2 with KOH, osmolality 290–300 mOsm/L) supplemented with 5 mM Tris-Succinate, 50 μg/mL digitonin and 1 μM Oregon Green 488 BAPTA calcium dye (Thermo Fisher Scientific). Excitation and emission wavelengths were 485/520 nm. Bolus injections of 5 μM CaCl_2_ were injected into each well every five minutes for 2 h. CRC was analyzed by calculating the amount of Ca^2+^ added to each well before a mitochondrial permeability transition (MPT) event was observed.

### ChIP-qPCR

Chromatin was isolated from cardiac tissue from TG mice that overexpress SMYD1a or from NRVMs that had been transduced with FLAG-tagged SMYD1a (MOI-25 for 48 h) for adenovirus-mediated overexpression, as previously described [20]. Cardiac tissue from WT mice or cells transduced with empty adenovirus was used as negative controls in these experiments. Chromatin immunoprecipitation was performed using the commercially available ChIP-IT High Sensitivity kit (Active Motif, 53,040) according to the manufacturer’s instructions. Chromatin-bound proteins were immunoprecipitated using Anti-H3K4me3 (Abcam, ab8580), Anti-H3K9me3 (Abcam, ab8898), and Anti-FLAG (Sigma Aldrich, F1804). Immunoprecipitated DNA was analyzed by qRT-PCR using primer sets that amplified the promoter region of *Ppargc1α* and an intergenic region as a negative control. qRT-PCR of each biological replicate was performed in duplicate with equal immunoprecipitated samples and input. Values were normalized to input measurements, and enrichment was calculated using ∆∆Ct method in Excel.

### Gene expression analysis

Real-time qPCR analysis was performed as described previously [20]. Briefly, total RNA was isolated using Trizol reagent (Thermo Fisher Scientific) according to the manufacturer’s instructions. cDNA was synthesized using Superscript III First-Strand Synthesis System (Life Technologies). Reactions were performed with 500 nM primers using SsoAdvanced Universal SYBR Green Supermix (Bio-Rad) per the manufacturer’s recommendations for run temperature and amplification time. Quantification of gene expression was performed on a Bio-Rad CFX Connect real-time PCR detection system. Analysis was performed using the 2^−ΔΔCt^ method, using *α-Actin* as a housekeeping gene. Primers were based on prior publications [20] or designed on NCBI Primer-BLAST and obtained from Thermo Fisher Scientific. A list of primers used in this study is provided in Supplemental Table S3.

### mtDNA quantification by qPCR

Genomic DNA for assessment of mitochondrial DNA (mtDNA) was isolated from 25 mg of cardiac tissue using a commercially available kit according to the manufacturer’s instructions (Qiagen, 69,504). Genomic DNA was added to a mixture of SYBR Green (Bio-Rad) and primers and analyzed with a Bio-Rad CFX Connect Real-Time system. Analysis of mtDNA/nDNA ratio was calculated by following the classical ∆∆Ct method used for qPCR analysis.

### Electrophoresis and western blotting

Western blotting analysis was performed as previously described [20]. Antibodies used in this study are as follows: anti-SMYD1 (Santa Cruz, SC2059 and Abcam, ab32482) Anti-FLAG (Sigma Aldrich F1804), Anti-SMYD2 (Abcam, ab108217), Anti-SMYD4 (Thermo Fisher Scientific, PA5-96,631), Anti-HSP90 (Cell Signaling Technology, 4874S), Anti-p53 (Abcam, ab131442), Anti-PGC-1α (Abcam, ab54481), Anti-Histone H3 (Abcam, ab1791), Anti-H3 tri methyl K4 (Abcam, ab8580), Anti-beta TUBULIN (ab6046), Anti-ACTIN (sc-1616), Anti-NDUFS3 (Abcam, ab196019), Anti-NDUFS2 (Abcam, ab110249), Anti-NDUFV1 (GeneTex, GTX102209), Anti-NDUFV2 (Proteintech, 15,301-1-AP), Anti-NDUFA9 (Abcam, ab14713), Anti-NDUFA10 (Santa Cruz, SC-376357), Anti-total OXPHOS cocktail (Abcam, ab110413) and Anti-VDAC1 (Abcam, ab14734), Anti-OPA1 (BD Biosciences, 612,606), Anti-MFN2 (Abcam, ab50843), Anti-Mitofilin (Proteintech, 10,179-1-AP).

### LC–MS/MS and proteomic analysis

Proteomic analysis was performed on ventricular tissue collected from transgenic and control mice at 1, 3, 6, 8 and 10 weeks of doxycycline diet and was analyzed using label-free quantitative LC–MS/MS, as described in our previous publications [18, 50]. Briefly, a total of 10 μg of protein lysate was prepared for filter-aided sample preparation. Samples were reduced, alkylated and digested with trypsin at a 1:40 ratio for 18 h at 37 °C. Tryptic peptides were separated by a reverse phase C-18 column using a 180 min multistep gradient and analyzed on an Orbitrap Velos Pro mass spectrometer interfaced with an Easy nlc-1000 UPLC [62]. Spectra were generated in data-dependent acquisition mode and peptides were fragmented with collision-induced dissociation. The top twenty MS1 peaks were analyzed at a resolution of 30,000 in rapid scan mode. Samples were run in duplicates to ensure sample reproducibility. Raw files were then processed with MaxQuant software (v 1.6.7.0) against the Uniprot *Mus musculus* database. Proteins were further filtered in Perseus (v 1.6.5.0) so only proteins with a minimum percentage of 70% in total have valid values. *T*-tests were performed in Perseus on filtered imputated data.

### Transmission electron microscopy (TEM) on cardiac tissue for mitochondria evaluation

Cardiac tissue samples were fixed in 2.5% glutaraldehyde, 1% paraformaldehyde, and 0.1 M sodium cacodylate buffer, overnight at 4 °C, following post-fixation for 1 h in 2% Osmium Tetroxide buffered with cacodylate buffer. After rinsing in filtered nano pure water, sections were stained with en-bloc stain in Uranyl Acetate for 1 h at room temperature. Following the post-staining, the tissues were dehydrated through a graded series of ethanol washes finishing with absolute acetone. Infiltrations were performed by incubating the specimens at room temperature in a gradually increasing concentration of Epoxy resin (Electron Microscopy Science, Hatfield, PA). Specimens were gradually transferred from 50% resin in acetone to 100% resin and embedded and polymerized at 60 °C for 48 h. Ultrathin sections (70 nm) were generated using a diamond knife (Diatome) with Leica UC 6 (Leica Microsystems, Vienna, Austria), and post-stained for 10 min with saturated uranyl acetate and for 5 min with Reinold stain. Sections were imaged at 120 kV with JEOL 1400 Plus and analyzed for mitochondria area and cristae morphology.

### BN-PAGE

For the assessment of Complex I organization, BN-PAGE was performed according to a previously published protocol [28]. Mitochondria isolated from transgenic and control cardiac tissue, or from cultured H9c2 cardiomyoblasts, in the amount of 50 μg were solubilized in 5% digitonin and lysates were resolved on NativePAGE Novex 3–12% gels (Thermo Fisher Scientific). After electrophoresis, in-gel Complex I activity was evaluated by incubating in Complex I activity substrate containing 2 mM Tris–HCl at pH 7.4, 1 mg/mL NADH and 2.5 mg/mL Nitrotetrazolium Blue chloride for 20 min, as previously described [8]. After identification of individual Complex I and Complex I–containing supercomplexes bands according to the in-gel activity, they were quantified using Image J and statistical differences were calculated from four (for cardiac tissue) or three (for cultured cells) independent measurements per gel and combined from independent electrophoresis runs.

### Citrate synthase activity assay

Citrate synthase (CS) activity was performed on isolated cardiac mitochondria using Citrate Synthase Activity Assay Kit (Sigma-Aldrich, MAK193), according to the manufacturer’s instructions. Briefly, 25 mg of cardiac tissue was minced on ice and homogenized using a glass dounce in mitochondria isolation buffer (210 mM mannitol, 70 mM sucrose, 5 mM HEPES and 1 mM EGTA at pH 7.2). Homogenate was then centrifuged at 400 × g for 7 min to remove the nuclear fraction and unbroken cells. Supernatant was subsequently centrifuged at 4000 × g for 30 min to isolate the crude mitochondrial fraction. Due to a very high CS activity of isolated mitochondria, the suspension for measurement was prepared to a final concentration of 5 mg/ml. Samples were mixed with the CS assay reaction mixes in a 96-well plate. The absorbance at 412 nm was measured continuously every 6 s for 5 min at room temperature using a Biotech Citation 5 plate reader. CS activity was calculated using a formula provided by the manufacturer.

### Statistical analysis

Unless specifically noted, data were collected from technical triplicates of three independent experiments and presented as the means with standard error of the mean (SEM). Statistical significance was evaluated using the unpaired two-tailed Student’s *t-*test or two-way ANOVA for pairwise comparisons. Asterisk * that indicates *p* < 0.05 was considered statistically significant.

## Results

### Expression of SMYD1 in failing heart

**Fig. 1:**
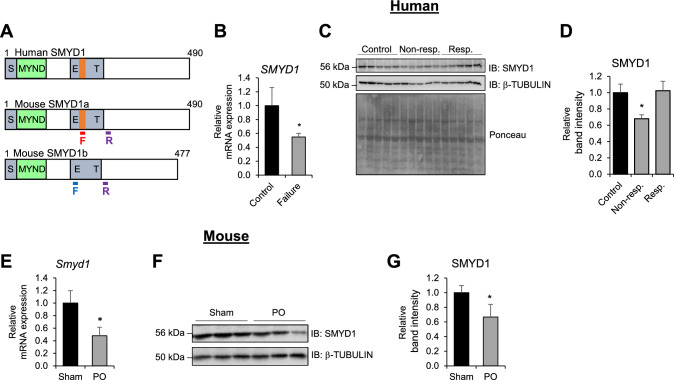
SMYD1 is downregulated in failing heart. **A** Transcript variants of SMYD1 in mouse and human hearts. Two splice variants of *Smyd1* in mouse heart differ by 13 amino acids inserted in *Smyd1a* (orange bar), which is conserved in the human transcript. Location of primers specific to either *Smyd1* isoform is indicated by underlined F (red and blue, below mouse *Smyd1a* and *Smyd1b* sequences, respectively) and R (purple). **B**–**D** Evaluation of SMYD1 expression in human hearts that underwent LVAD treatment by **B** qRT-PCR and **C**, **D** western blot (WB). Non-resp. indicates non-responders and Resp. indicates responders. Asterisk * indicates *p* < 0.05, *n* = 5/Control, *n* = 10/Failure (qRT-PCR), and *n* = 5/group (WB). **E**–**G** qRT-PCR (**E**) and western blot evaluation (**F**) with quantification (**G**) of SMYD1 levels in mouse hearts 5 weeks after permanent occlusion (PO) of the LAD or Sham surgery. Asterisk * indicates *p* < 0.05, *n* = 6–7

SMYD1, the founding member of the SMYD family, is a myocyte-specific lysine methyltransferase that has been shown to regulate transcription by methylating histone H3 lysine K4 [51, 57]. While all five members of the SMYD family are expressed in mammals, only SMYD1 is restricted to striated muscle [32, 57]. In humans, the *SMYD1* gene produces a single transcript that shares high sequence homology and striated muscle specificity with two murine transcript variants: isoform 1 (GenBank: NM_001160127.1, referred to as *Smyd1a)* and isoform 2 (GenBank: NM_009762.2, referred to as *Smyd1b*), as shown in Fig. 1A [57]. Specifically, mouse SMYD1a shares 94% sequence homology with human SMYD1, making it the mouse ortholog of human SMYD1 [57]. Knowing that SMYD1 plays a significant role in cardiac function, we became interested in evaluating SMYD1 expression levels in human heart failure patients. We obtained samples from patients with advanced heart failure who were prospectively enrolled at the time of implantation of a left ventricular assist device (LVAD) as a bridge-to-transplant or destination therapy [3]. These samples were evaluated at the time of implantation and the clinical characteristics of study subjects (heart failure) and healthy donors (control) are presented in Supplementary Table S1 and S2, respectively. Interestingly, SMYD1 was significantly decreased in cardiac tissue collected from patients diagnosed with end-stage heart failure at the time of LVAD implantation (Fig. 1B). This is consistent with two reports published recently, where SMYD1 transcript levels were downregulated in cardiac tissue obtained from patients with ischemic or non-ischemic cardiomyopathy undergoing LVAD implantation [65] or from left ventricular tissue samples obtained at the time of heart transplantation [53]. However, when these heart failure patient samples were further stratified into two categories, which described their response to LVAD unloading, the results were intriguing. Specifically, the samples were categorized as either responders or non-responders using left ventricular ejection fraction (LVEF) and left ventricular end-diastolic diameter (LVEDD) measurements during diminished LVAD support “turn-down” echocardiograms. Responders were defined as patients with a LVEF > 40% and LVEDD ≤ 5.9 cm at six months post LVAD implantation, whereas non-responders were defined as patients with an LVEF < 35% and with < 50% relative improvement in LVEF regardless of the final LVEDD. We performed protein expression analysis of these samples and observed that while SMYD1 was significantly downregulated in patients who did not respond to LVAD therapy (non-responders), its expression was not reduced in patients whose heart function significantly improved after LVAD implantation (responders), when compared to cardiac tissue from healthy donor hearts (Fig. 1C, D). This suggests that higher levels of SMYD1 may be beneficial for the heart.

Next, quantified SMYD1 expression in a mouse model of ischemic injury as this has never been examined before. Our results showed that SMYD1 gene and protein expression in mice that were subjected to permanent occlusion (PO) of the LAD was significantly decreased 5 weeks after the surgery (Fig. 1E–G).

### Generation and validation of *Smyd1a* transgenic mice

**Fig. 2:**
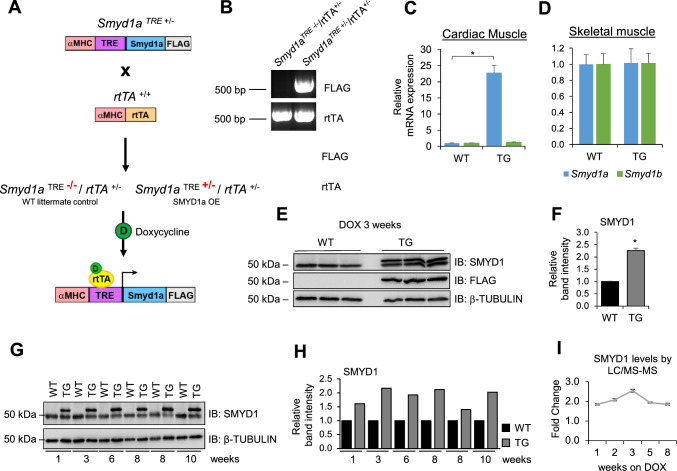
Generation of inducible, cardiomyocyte-specific SMYD1a overexpression in mice. **A** Transgenic mice were generated by crossing animals expressing a reverse tetracycline-controlled transactivator (rtTA) under the *αMHC* promoter with mice carrying the *Smyd1a*-FLAG gene under the modified *αMHC* promoter containing a TRE element. These mice express *Smyd1a*-FLAG only upon doxycycline (D) administration (80 mg/kg/day). **B** Genotyping of mice carrying the *Smyd1a*-FLAG and *rtTA* alleles. Doxycycline (DOX) induced expression of mouse SMYD1 isoforms was confirmed by RT-qPCR in **C** cardiac and **D** skeletal muscle showing overexpression specific to the mouse heart. Asterisk * indicates *p* < 0.05, *n* = 3–4. Western blotting (**E**) and quantification (**F**) of SMYD1 in mouse cardiac tissue 3 weeks after doxycycline treatment. Asterisk * indicates *p* < 0.05, *n* = 3. Long-term doxycycline treatment maintains SMYD1 expression at ~ twofold increase in transgenic (TG) mice as compared to wild type (WT) mice, as shown by **G** representative western blotting (quantified in **H**), and **I** LC/MS–MS, *n* = 3–4

We have previously shown that inducible cardiomyocyte-specific knockout of *Smyd1* in mice leads to pathological organ remodeling, chamber dilation and heart failure [20]. Thus, we became interested in evaluating SMYD1 gain-of-function under basal conditions and after stress in an animal model. We generated transgenic mice (TG) capable of inducible, cardiomyocyte-specific overexpression of the mouse ortholog of human SMYD1, which is SMYD1a, by crossing animals expressing a reverse tetracycline-controlled trans activator under the *αMHC* promoter (αMHC-rtTA) with mice carrying the *Smyd1a* gene (tagged with FLAG on the C-terminus) under the modified *αMHC* promoter containing a TRE (7 tet-o repeats) element. These mice express SMYD1a-FLAG upon doxycycline (DOX) administration. The experimental scheme for generating these mice is shown in Fig. 2A, and the genotyping of these alleles is confirmed by PCR in Fig. 2B. The resulting mice were *Smyd1a*^TRE+/−^/*rtTA*^+/−^, referred to as transgenic (TG) or SMYD1a-OE, and *Smyd1a*^TRE−/−^/*rtTA*^+/−^ referred to as WT littermate controls. TG mice develop, grow and reproduce normally. These mice are fed DOX-containing chow to induce expression of SMYD1a-FLAG specifically in the heart (Fig. 2C), while skeletal muscle SMYD1 remained unaffected (Fig. 2D). We also confirmed SMYD1a overexpression by western blotting (Fig. 2E–H) as well as LC/MS–MS (Fig. 2I) and found that long-term DOX treatment of these mice allows for consistent ~ twofold overexpression of SMYD1a. It is important to note that SMYD1a-FLAG appears on western blots as a slightly higher molecular weight band than endogenous SMYD1a.

### SMYD1a overexpression in mice protects from ischemic injury

**Fig. 3:**
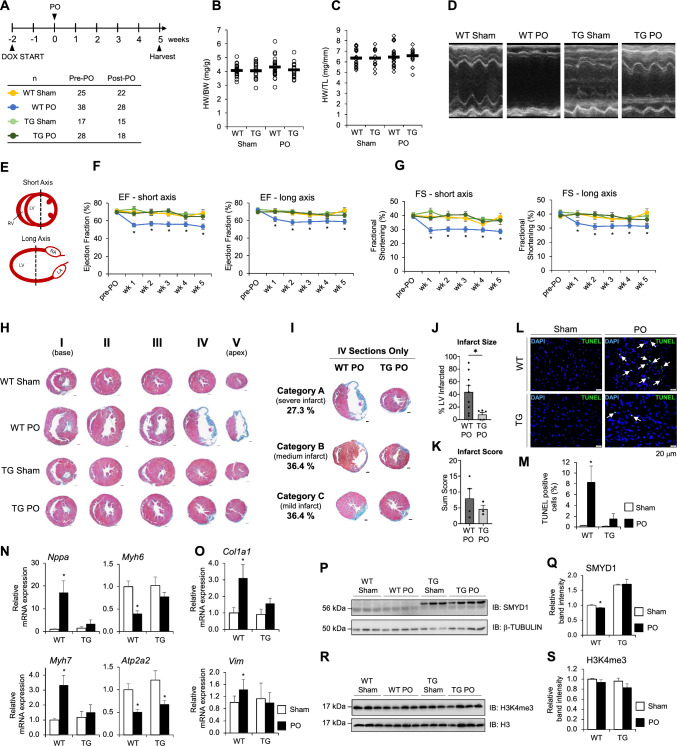
SMYD1a overexpression protects the heart from ischemic injury. **A** Mice were fed doxycycline chow for 2 weeks, subjected to permanent occlusion (PO) of the LAD, monitored weekly by echocardiography and hearts were harvested at 5 weeks after PO surgery. Biological replicates for phenotyping studies are presented in the table. **B** Heart weight: body weight and** C** heart weight: tibia length ratios show no significant changes between experimental groups. **D** Cardiac function was measured by echocardiography and represented by **D** echocardiographs and measured **E** at the view of the middle point of the short or long axis from M-mode to determine **F** ejection fraction and **G** fractional shortening. Echo data show preserved cardiac function in transgenic (TG) mice when compared to littermate controls (WT) that underwent PO surgery. Asterisk * indicates *p* < 0.05. **H** Five serial sections of the hearts were acquired at 5 weeks from the base (I) to apex (V) and these representative images show that SMYD1a mitigates ischemic injury in TG mice compared to WT control which lack the *rtTA* transgene and are also maintained on DOX chow. Bar = 100 mm. **I** The ischemic injury from this surgical model was quantified by categorizing all IV sections into severe, medium, and mild infarct categories and representative images further confirm a reduction in infarct size in TG mice. Bar = 100 mm.** J** Infarct size was quantified in Masson’s trichrome-stained sections by measuring the midline length of injury as a percentage of total midline left ventricle circumference. Asterisk * indicates *p* < 0.05, *n* = 5–9. **K** The presence and extend of infarct lesions were scored using H&E-stained slides; scores were summed for each animal, n = 3–4. **L** Apoptosis in cardiac tissue harvested 48 h after PO of the LAD was detected by TUNEL assay and TUNEL positive cells (green, pointed with arrows) were quantified (**M**) in all experimental groups indicating a significant decrease in apoptosis in TG PO mice. Asterisk * indicates *p* < 0.05, *n* = 9–15. **N** Fetal genes: *Nppa*, *Myh6*, *Myh7* and *Atp2a2*, and **O** fibrotic genes: *Vim* and *Col1a1* were measured by qRT-PCR and values expressed as relative mRNA intensities relative to WT control mice. Asterisk * indicates *p* < 0.05, *n* = 11–19. **P**,** Q** Permanent occlusion had no effect on the SMYD1 expression levels in TG mice. *N* = 3–4. **R**, **S** Neither overexpression of SMYD1a nor permanent occlusion affected global levels of histone H3K4 trimethylation as shown by **R** western blotting and **S** quantification. Asterisk * indicates *p* < 0.05, *n* = 3–4

To assess the physiological effects of SMYD1a gain-of-function and evaluate if SMYD1a can attenuate disease-induced remodeling in an animal model, we subjected TG mice and their littermate controls (WT) to permanent occlusion (PO) of the LAD (or Sham) surgery. Mice were fed chow-containing doxycycline for 2 weeks prior to surgeries to induce overexpression of SMYD1a protein and were monitored weekly by echocardiography (Fig. 3A). Analysis of heart weight-body weight ratios (HW/BW) as well as tibia length-body weight (TL/BW) ratios 5 weeks after PO surgery revealed that there are no significant differences between WT and TG mice in response to LAD ligation (Fig. 3B, C). We believe this is due to the combined effects of hypertrophic growth and cell death occurring at the same time but in different regions of the heart. Interestingly, our TG mice subjected to PO showed no decrease in ejection fraction (% EF) and fractional shortening (% FS) by echocardiography when compared to WT animals, which displayed a significant decrease in these measurements (Fig. 3D–G). Our histological analysis showed reduced infarct size in cardiac tissue from TG mice (Fig. 3H). For this analysis, we defined three qualitative categories of damage in PO hearts to evaluate the variability of ischemic injury (Fig. 3H). Category A, observed in 27.3% of WT PO hearts, presented a severe phenotype, with more than half of the left ventricle forming a fibrotic scar; category B, observed in 36.4% of WT PO hearts presented a medium phenotype with equal amounts of fibrosis and cardiomyocytes in the left ventricle; and category C, observed in 36.4% of WT PO hearts presented a mild infarct, having a relatively small fibrotic scar with the majority of the left ventricle tissue remaining viable. However, when we examined the severity of infarct in TG PO mice, these animals showed little variability with 100% of samples characterized by a mild (category C) infarct. We have still included three representative images (Fig. 3I) for these TG PO mice to highlight the lack of variability within this sample cohort. Furthermore, we evaluated the infarct size by first scoring tissue sections according to the extent of the lesioned area [22] and then measuring the midline infarct lengths [55] and showed a significant decrease in infarct size in TG PO mice as compared to WT PO group (Fig. 3J, K). In addition, we evaluated the level of apoptosis in transgenic mice which overexpress SMYD1a or in littermate controls (WT) 48 h after permanent occlusion of the LAD or Sham surgery by TUNEL assay. Our results of the tissue sections adjacent to the infarct area revealed a significant decrease in TUNEL-positive cells in TG mice in response to ischemic injury (Fig. 3L, M and Supplementary Fig. S1A). To confirm that the observed effect is cardiomyocyte-specific, cells were co-stained with cardiac troponin C (Supplementary Fig. S1A). In addition, cultured H9c2 cardiomyoblasts that overexpress SMYD1a and were subjected to hypoxic conditions showed a significant decrease in TUNEL-positive cells (Supplementary Fig S1B, C). Evaluation of markers of angiogenesis showed that overexpression of SMYD1a has no effect on the expression of *Vegfa* and *Fgf*-2 in TG mice and 48 h after permanent occlusion (Supplementary Fig. S2). Overall, this data suggests that overexpression of SMYD1a protects from apoptosis in the ischemic heart.

Because overexpression of SMYD1a protects the heart from ischemic injury we investigated the expression of genes commonly dysregulated during cardiac disease in TG mice that were subjected to PO surgery [20, 47]. The gene expression analysis of these transcripts: *Nppa*, *Myh6*, *Myh7* and *Atpa2a2* (Fig. 3N) as well as markers associated with fibrosis: *Vim, Col1a* (Fig. 3O) revealed significant changes following overexpression of SMYD1a. In this data, we saw two key observations: first, we did not observe any significant changes in these transcripts at basal levels in WT and TG mice. Second, gene expression changes that are hallmarks of hypertrophy and failure in mice (i.e. increased *Nppa*) were unaffected in TG mice after PO, suggesting that overexpression of SMYD1a inhibits disease-induced transcriptional changes (Fig. 3N, O).

Evaluation of SMYD1 protein expression in TG mice subjected to permanent occlusion of the LAD showed similar levels of SMYD1 in TG mice 5 weeks after PO surgery (Fig. 3P, Q). Although there is no evidence to support interactions or molecular compensation between SMYD1a and other SMYD family members, we examined their expression in our TG mice at basal levels and after PO. Our analysis revealed that SMYD1a overexpression does not affect the transcript or protein expression levels of other SMYD family members, and only the *Smyd3* transcript showed a modest decrease in response to permanent occlusion (Supplementary Fig. S3A, B). In addition, we also examined the expression of two proteins (p53 and HSP90) that have been shown to interact with SMYD1 and only HSP90 was increased in TG mice (Supplementary Fig. S3B).

Finally, it was previously shown that SMYD1 trimethylates lysine K4 on histone H3 [56], therefore we investigated whether overexpression of SMYD1a affects global levels of this histone post-translational mark. We performed western blotting for H3K4me3 in total cell lysates collected from TG and WT mice after PO or Sham surgeries and determined that overexpression of SMYD1a had no effect on global levels of histone H3K4me3 (Fig. 3R, S). While we expect that histone H3K4me3 may still be regulated by SMYD1a in this model at the individual gene level, we do not see changes in this post-translational mark globally. Methylation of histone H3K4 is a very dynamic process regulated by multiple methyltransferases and demethylases [14, 54], therefore, future work will be needed to understand the complete repertoire of enzymes that regulate this mark in the heart.

### SMYD1a overexpression enhances mitochondrial respiration and ATP production

**Fig. 4:**
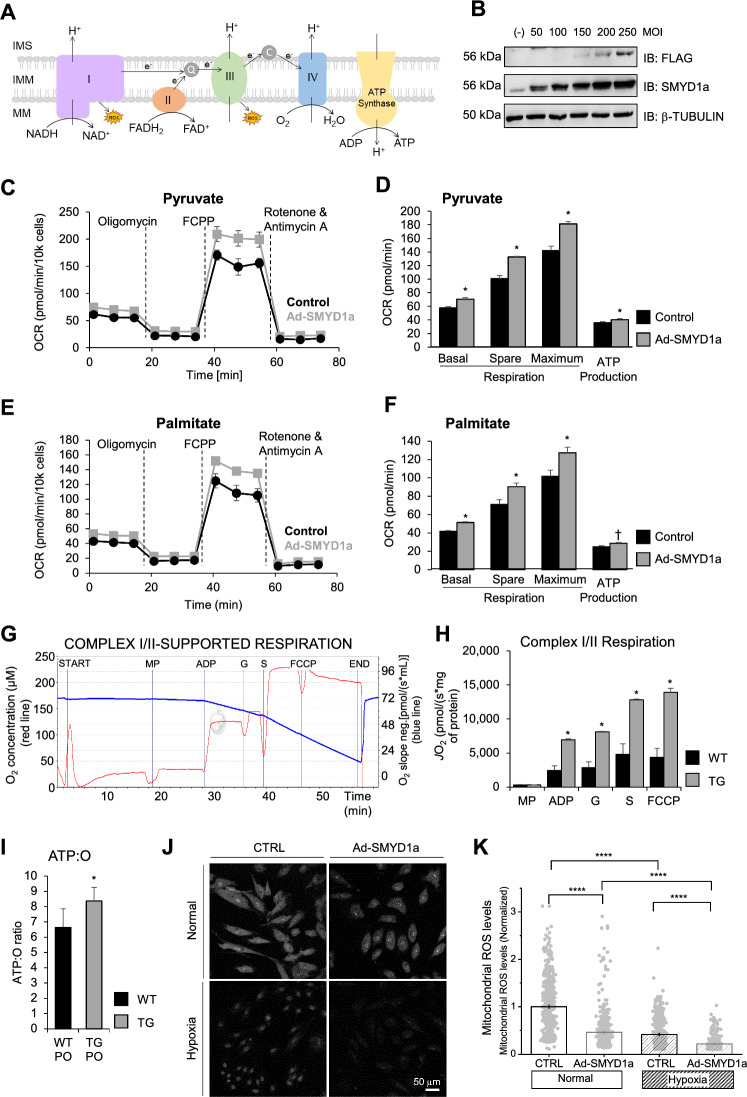
SMYD1a overexpression enhances mitochondrial respiration and lowers ROS production. **A** Model of the mitochondrial electron transport chain (ETC) and ATP synthesis. *IMS* intermembrane space, *IM* inner mitochondrial membrane, *MM* mitochondrial matrix. **B** Western blotting showing expression levels of SMYD1a in H9c2 cardiomyoblasts that were transduced with Ad-SMYD1a-FLAG. **C**–**F** Cell Mito Stress Test was conducted using a Seahorse Bioscience XFe96 analyzer by sequentially injecting 1 mM oligomycin, 5 mM FCCP, and 1 mM rotenone + antymycin A inhibitors. H9c2 cardiomyoblasts were transduced with Ad-SMYD1a and oxygen consumption rates (OCR) were recorded and quantified in the presence of **C**, **D** pyruvate or **E**, **F** palmitate as substrates. Quantitative analysis (**D**, **F**) of mitochondrial OCR from H9c2 cells overexpressing SMYD1a indicates significant increases in respiration in response to various inhibitors for both substrates. Asterisk * indicates *p* < 0.05, † indicates *p* = 0.08, *n* = 5. **G** Representative respiration traces in the Complex I/II-linked OXPHOS state detected by Oroboros O2k oxygraph and quantitative analysis (**H**) of mitochondrial OCR from TG and WT control groups showing increased oxygen consumption in TG mice as compared to WT controls. Malate-pyruvate (MP), adenosine diphosphate (ADP), glutamate (G), succinate (S), carbonyl cyanide m-chlorophenyl hydrazone (FCCP) were used as substrates. Asterisk * indicates *p* < 0.05, *n* = 4. **I** Evaluation of ATP production rates in isolated mitochondria from TG and WT mice subjected to PO show that molar amounts of ATP produced per mole of atomic oxygen consumed (known as ATP:O ratio) is increased in TG mice 24 h after PO. Asterisk * indicates *p* < 0.05, *n* = 5–6. **J**, **K** ROS levels are lower in H9c2 cardiomyoblasts that overexpress SMYD1a, and in hypoxic conditions, as measured by relative MitoSOX fluorescence and quantified by normalization to control. Asterisk **** indicates *p* < 0.0001, *n* = 353–622

The electron transport chain is a cluster of four protein complexes in mitochondria that transfer protons through a membrane and drive the synthesis of ATP needed for essential cellular processes (Fig. 4A). The mammalian heart requires an enormous amount of ATP and deficits in ATP production have been linked to the development of heart failure and ischemic injury [40]. Thus, to investigate the effects of SMYD1a overexpression on the mitochondrial function we performed a Cell Mito Stress Test in H9c2 cardiomyoblasts transduced with adenovirus carrying SMYD1a-FLAG (Fig. 4B). We observed that overexpression of SMYD1a led to an increase in basal, spare, and maximal respiration capacity as well as increased ATP production when glucose or palmitate was used as the major respiration substrate (Fig. 4C–F). Next, using an Oroboros O2k oxygraph we performed high-resolution mitochondrial respirometry on mitochondria isolated from our transgenic and WT mice. Consistent with data in isolated cells, we show that SMYD1a overexpression leads to ~ 2–3-fold increase in mitochondrial respiration through Complex I and II (Fig. 4G, H). This data represents maximal State-3 respiration, which demonstrates the rate of mitochondrial respiration and ATP synthesis at their maximum pull from supraphysiological conditions. Because it is simulated to test the mitochondria at their maximum respiratory capacity, it does not necessarily reflect the absolute value of in vivo physiological rates. Overall, this data shows that SMYD1a enhances mitochondrial oxidative capacity through carbohydrate-supported-ATP production. It is important to note that there are significant differences in basal expression of SMYD1 between cultured H9c2 cardiomyoblasts and cardiac tissue (Supplementary Fig. S4A-C). Thus, to demonstrate that the overexpression of SMYD1a in H9c2 cells is comparable to levels in TG mice we tested increasing MOI of adenovirus on SMYD1a protein levels (Supplementary Fig. S4D, E) and oxygen consumption rates as measured in Cell Mito Stress Test (Supplementary Fig. S4F-G). This data shows that when H9c2 cardiomyoblasts express SMYD1a at the same levels seen in adult cardiac tissue they display the same response to oxygen consumption and mitochondrial respiration seen in TG mice.

It has been previously reported that the failing heart loses its metabolic flexibility and can become energy-deficient because of a decrease in its ability to produce ATP [40]. To further assess oxidative phosphorylation (OXPHOS) efficiency in mitochondria isolated from WT and TG mice subjected to permanent occlusion of the LAD we evaluated ATP production rates 24 h after PO by measuring molar amounts of ATP produced per mole of atomic oxygen consumed, known as the ATP:O ratio [35]. Interestingly, we observed an increase in the ATP:O ratio in TG PO mice, indicating they have a higher respiratory efficiency (Fig. 4I). This enhanced respiratory efficiency would be particularly important during ischemic injury considering the oxygen supply would be limited in cardiac tissue.

The natural by-product of electron transport chain activity in mitochondria is the production of reactive oxygen species, or ROS (Fig. 4A) [4, 68]. Because we see a significant increase in mitochondrial respiration in our TG mice, we investigated the effects of SMYD1a overexpression on ROS production. Cultured H9c2 cardiomyoblasts overexpressing SMYD1a were subjected to hypoxic conditions and imaged after staining with ROS-sensitive MitoSOX dye (Fig. 4J). The results show lower ROS production in cells that overexpress SMYD1a and after induced hypoxia (Fig. 4K).

### SMYD1a overexpression maintains metabolic homeostasis by regulating PGC-1α and its downstream targets

**Fig. 5:**
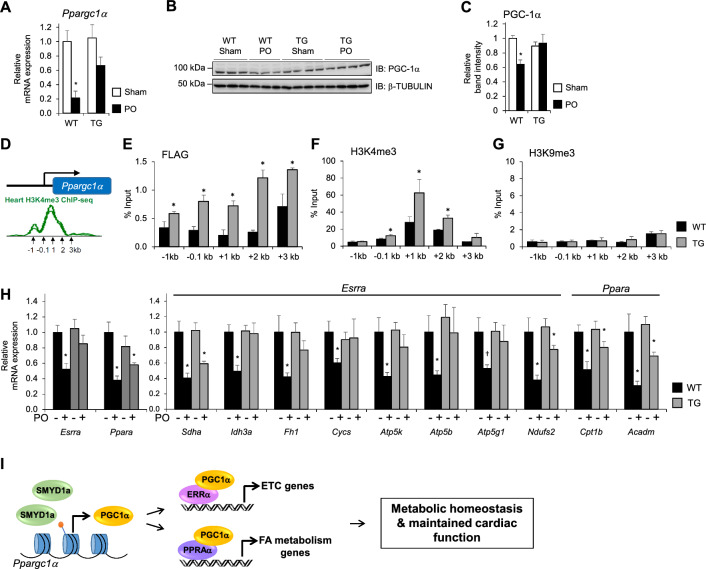
SMYD1a overexpression prevents a decline in metabolic enzymes downstream of PGC-1a. **A** Expression of PGC-1a was quantified via **A** qPCR and **B**, **C** western blotting in cardiac tissue from TG and WT mice 48 h after permanent occlusion of the LAD or Sham surgeries. These data show that PGC-1α expression is significantly decreased in response to ischemic stress (PO), however, overexpression of SMYD1a is capable of maintaining PGC-1α at basal levels. Asterisk * indicates *p* < 0.05, *n* = 3–6. **D** Publicly available ChIP-Seq data for histone H3K4me3 at the *Ppargc1α* promoter was used to design primers to use in ChIP-qPCR for SMYD1a-FLAG, H3K4me3 and H3K9me3 in cardiac tissue from TG and WT mice showing **E** SMYD1a binding at the *Ppargc1α* promoter which **F** increases trimethylation of histone H3K4 under basal conditions. **G** H3K9me3 ChIP was used as a negative control. Asterisk * indicates *p* < 0.05, *n* = 4–6. **H** Overexpression of SMYD1a rescues expression of genes involved in OXPHOS and fatty acid oxidation which are regulated by ERRα or PPARα transcription factors, respectively, and downregulated in response to ischemic injury. Asterisk * indicates *p* < 0.05, † indicates *p* < 0.08, *n* = 4–6. **I** Under basal conditions, SMYD1a, through its histone methyltransferase activity, regulates the expression of PGC-1α and its downstream targets which results in maintained metabolic homeostasis and cardiac function

We have previously shown that SMYD1 can regulate mitochondrial respiration in cultured cells, at least in part, by modulating the expression of PGC-1α, a master regulator of mitochondrial energetics. We also demonstrated that loss of *Smyd1* in the adult mouse heart leads to reduction of histone H3K4 trimethylation at the *Ppargc1α* promoter [62], however, we have never examined direct binding or methylation of the *Ppargc1α* promoter by SMYD1 in vivo. Therefore, in this study we first quantified expression of PGC-1α via qPCR and western blotting in cardiac tissue from transgenic (TG) and control (WT) mice 48 h after permanent occlusion and show that at this early time point PGC-1α levels are decreased (Fig. 5A–C), consistent with previous reports [5]. Interestingly, however, increased expression of SMYD1a maintains PGC-1α expression at normal levels in our transgenic mice (Fig. 5A–C) but does not elevate it above basal levels. We believe that this is due to the tightly controlled regulation of this gene and reflects not only the positive regulators of expression but also the homeostatic balance of negative regulators, e.g. demethylases.

To determine whether the regulation of PGC-1α expression occurs in this model through direct binding of SMYD1a to the *Ppargc1α* promoter we performed chromatin immunoprecipitation of FLAG-tagged SMYD1a, as previously published [20, 62]. Immunoprecipitated DNA was analyzed by ChIP-qPCR using primers that amplify the promoter region of *Ppargc1α* (Fig. 5D). For the first time in an animal model, we demonstrate that SMYD1a binds to the *Ppargc1α* promoter and increases trimethylation of histone H3K4 (Fig. 5E–G). Additionally, we confirmed this in isolated neonatal rat ventricular myocytes (NRVMs) via ChIP-qPCR by transducing cells with adenovirus carrying SMYD1a-FLAG and observed binding and hypermethylation (H3K4me3) of the *Ppargc1α* promoter (Supplementary Fig. S5A-C).

PGC-1α induces expression of metabolic genes through the coactivation of two key transcription factors, ERRα and PPARα, that directly regulate genes involved in fatty acid metabolism (through PPARα) or the TCA cycle and electron transport chain (through ERRα) [5, 62]. Therefore, we also examined the expression of these downstream targets of PGC-1α in TG and WT mice. Our data show that while the genes involved in OXPHOS and fatty acid oxidation are downregulated in response to ischemic injury, overexpression of SMYD1a maintains their expression at basal levels, including *Sdha, Idh3a, Fh1, Cycs, Atp5k, Atp5b, Atp5g1, Ndufs2, Cpt1b, Acadm* (Fig. 5H).

Overall, this data provide key insights into SMYD1a’s regulation of PGC-1α and confirm that maintaining PGC-1α expression is necessary for metabolic homeostasis (Fig. 5I), however, it does not account for the increase in oxygen consumption in our SMYD1a transgenic mice. Therefore, we were interested in further examining key components of cardiac respiration in this model to identify the driver(s) of enhanced mitochondrial respiratory capacity.

### SMYD1a regulates ETC supercomplex formation and cristae morphology

**Fig. 6:**
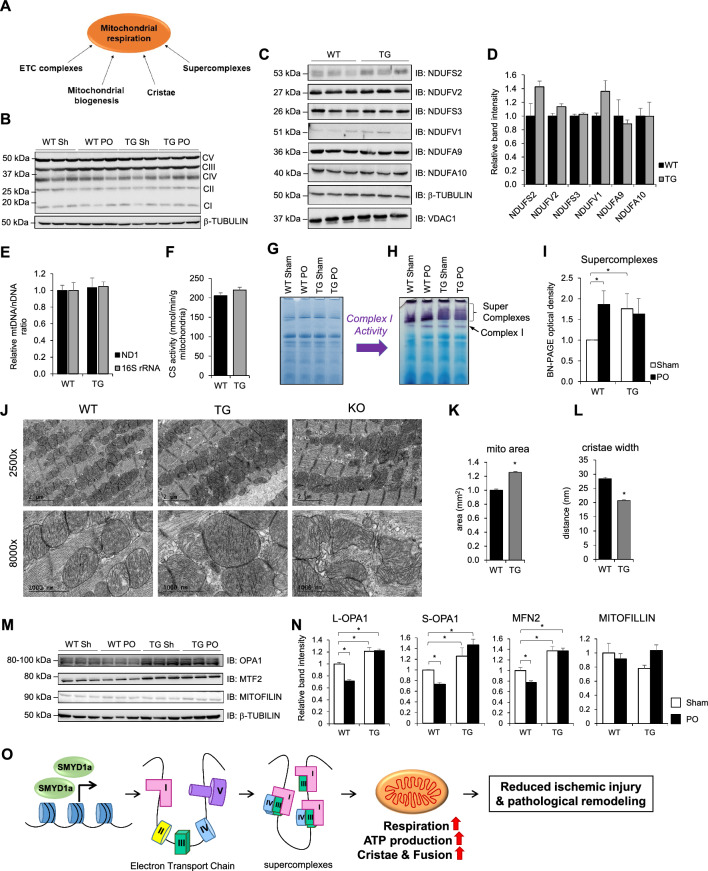
SMYD1a overexpression regulates mitochondrial respiration by increasing the formation of supercomplexes and cristae. **A** Diagram showing factors influencing mitochondrial respiration. **B** Western blotting evaluation of the electron transport chain (ETC) complex subunits using an antibody cocktail showing no significant changes in abundance of ETC complexes in TG mice or after PO, *n* = 3. **C** Immunoblotting for additional ETC subunits showed **D** modest changes in a few subunits (NDUFS2, NDUFV1), but no change in most of those examined, *n* = 3. **E**–**F** Mitochondrial biogenesis is absent in transgenic SMYD1a mice as confirmed by three markers: **E** mitochondrial DNA quantified by ND1 and 16S rRNA via qPCR, *n* = 6, and **F** citrate synthase activity in TG and WT mice which showed no change after 2 weeks of DOX-induced SMYD1a overexpression, *n* = 3–4. **G**–**I** SMYD1a overexpression increases supercomplex formation as determined by blue-native PAGE gel stained with **G** Coomassie, evaluated for **H** Complex I activity and **I** quantified in WT and TG mice 48 h after permanent occlusion of the LAD or Sham surgery. Asterisk * indicates *p* < 0.05, *n* = 3–4. **J** Electron micrographs of cardiac tissue from WT, SMYD1a transgenic mice and *Smyd1* KO mice show that while loss of SMYD1 leads to loss of cristae structure, SMYD1a overexpression leads to **K** larger mitochondria with **L** more dense, narrower cristae. Asterisk * indicates *p*-value *p* < 0.05, *n* = 3–7. **M,N**) Western blotting and quantification analysis showing increased expression of OPA1 and MFN2 and no change in mitofillin expression in TG mice, as compared to WT controls. Asterisk * indicates *p* < 0.05, *n* = 3. **O** SMYD1a overexpression leads to the formation of ETC supercomplexes and more dense and narrower cristae that results in increased mitochondrial respiration and ATP production through which it ultimately reduces ischemic injury and pathological remodeling

Mitochondrial respiration is the most important generator of energy in the cell. Abnormalities in this process lead to a variety of inherited and acquired cardiovascular diseases including ischemic heart disease. Several factors have been identified which positively influence mitochondrial respiratory capacity, including Electron Transport Chain (ETC) Complexes [24, 45, 67], cristae morphology [21, 26, 44], mitochondrial biogenesis, and ETC supercomplexes [27, 34] (Fig. 6A). To further examine the mitochondrial function and elucidate the basis for the enhanced respiration in our TG mice, we systematically evaluated those components that positively affect respiration. First, we evaluated the abundance of the ETC complex subunits using an antibody cocktail containing one antibody for each of the five complexes and found no difference in our TG mice before or after PO (Fig. 6B). Additional immunoblotting of other ETC subunits showed no statistical difference in expression in TG mice (Fig. 6C, D).

To evaluate mitochondrial biogenesis in TG mice, we quantified three markers: 16S rRNA and ND1, which are encoded by mitochondrial DNA, and citrate synthase activity and showed no change in SMYD1a overexpression mice (Fig. 6E, F). This is consistent with our high-resolution respirometry measurements, in Fig. 4, which were normalized to total mitochondrial content, suggesting that the increased respiration is independent of mitochondrial content.

In response to a cell’s energy demands, a certain portion of respiratory chain complexes can form higher-order structures called ‘supercomplexes’. These supercomplexes form within the folds of the inner mitochondrial membrane (known as cristae) and enhance respiration efficiency [11]. Indeed, increased cristae formation has been shown to increase supercomplex formation [11, 12]. Therefore, to evaluate the formation of electron transport chain supercomplexes, we performed blue-native PAGE of mitochondria isolated from TG and WT mice and showed increased supercomplex formation in transgenic mice at basal levels (Fig. 6G–I). The presence of these supercomplexes prior to ischemia may protect cardiac tissue from ischemic injury. The formation of ETC supercomplexes also increased 48 h after ischemic injury in WT animals, which we believe is part of a compensatory mechanism in response to the metabolic demands of the tissue. In addition, we performed BN-PAGE of mitochondria isolated from H9c2 cardiomyoblasts that either overexpressed or lacked SMYD1a and showed that overexpression of SMYD1a can increase the formation of supercomplexes while loss of *Smyd1* has no effect (Supplementary Fig. 6A, B). Next, we looked at mitochondrial morphology via electron microscopy of cardiac tissue (Fig. 6J–L). Overexpression of SMYD1a resulted in larger mitochondria (Fig. 6J, K) with more dense, narrow cristae (Fig. 6L) as compared to WT controls, while loss of SMYD1 in *Smyd1*-KO mice (Supplementary Fig. S7A, B) resulted in abnormal mitochondria with loss of cristae structure (Fig. 6J). We next analyzed the expression of proteins that regulate cristae morphology and mitochondria fusion. Our western blot analysis showed that the known cristae remodeler OPA1 as well as the mitochondrial fusion protein MFN2 are upregulated in TG mice and after PO, but not mitofilin (Fig. 6M, N). We also analyzed mitochondrial Ca^2+^ and showed that overexpression of SMYD1a increased basal mitochondrial calcium but did little to alter calcium levels under hypoxic conditions. We observed that the increases in calcium levels were much lower in cells that overexpressed SMYD1a suggesting less calcium is taken up into the mitochondria during ischemia (Supplementary Fig. S8). Overall, this data suggests that SMYD1a overexpression protects the myocardium from ischemic injury by enhancing mitochondrial respiration and ATP production through the remodeling of cristae and formation of electron transport chain supercomplexes (Fig. 6O).

### SMYD1a regulates OPA1 expression to enhance mitochondrial respiration

**Fig. 7:**
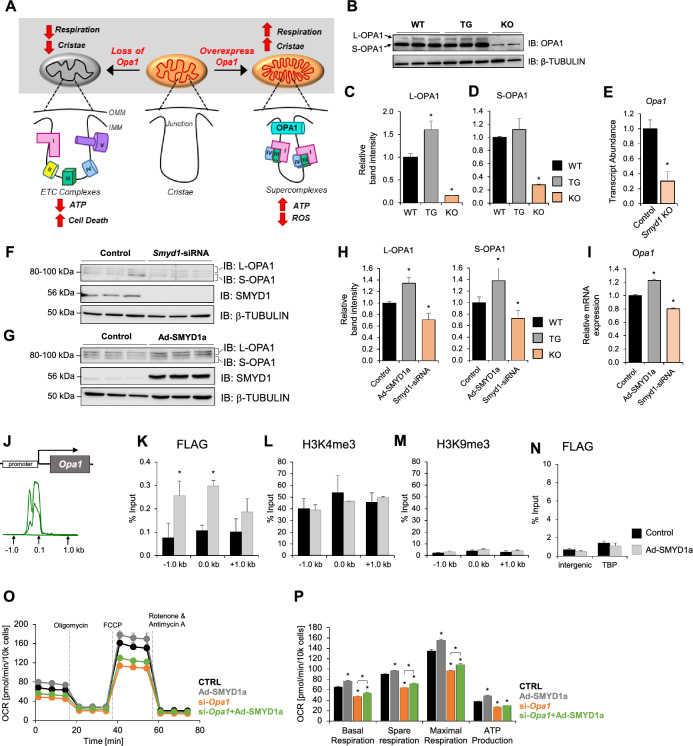
SMYD1a regulates OPA1 expression in the cardiomyocyte. **A** OPA1 oligomers bind the inner mitochondrial membrane (IMM) at cristae junctions and regulate cristae structure. Overexpression of OPA1 has been shown to alter cristae morphology leading to reduced cristae width, increased cristae number and enhanced respiration by stabilizing supercomplex formation (right). Conversely, loss of OPA1 leads to reduced respiration and ATP, loss of cristae structure and cell death (left). **B**–**D** Western blotting analysis showing changes in OPA1 expression which is **B**, **C** increased in SMYD1a TG mice and **B**, **D** decreased in *Smyd1* knockout (KO) mice at the **D** protein and **E** transcript level. Asterisk *indicates *p* < 0.05, *n* = 2–3. **F**–**I** Cultured H9c2 cardiomyoblasts were transfected with **F** either scr-siRNA (control) or *Smyd1*-siRNA for 72 h or **G** transduced with either empty virus (Control) or SMYD1a-FLAG adenovirus (Ad-SMYD1a) for 72 h. Both **H** wester blotting and **I** qPCR analysis show that siRNA-mediated *Smyd1* knockdown in H9c2 cells led to significant downregulation of OPA1 (**F**, **H**, **I**) whereas overexpression of SMYD1a increased OPA1 expression (**G**, **H**, **I**). Asterisk * indicates *p* < 0.05, *n* = 3. **J** Previous ChIP-Seq studies provided enrichment data for histone H3K4me3 at the *Opa1* promoter which was used to design primers for chromatin immunoprecipitation (ChIP) and qPCR. ChIP-qPCR for adenovirus-mediated SMYD1a overexpression (using FLAG antibody) show **K** enrichment in the promoter region of *Opa1*; however, **L** no corresponding enrichment of histone H3 lysine K4 trimethylation was detected in these regions. **M** Histone H3K9me3 and **N** random intergenic and control *Tbp* promoter were used as negative controls to show that SMYD1 is not enriched by ChIP-qPCR at either of these regions. Asterisk * indicates *p* < 0.05, *n* = 4–6. **O**, **P** Overexpression of SMYD1a partially rescues the downregulation of cellular respiration caused by silencing of *Opa1*, as shown by the Cell Stress Test in H9c2 cardiomyoblasts. Asterisk * indicates *p* < 0.05, *n* = 4–6

It has been previously reported that mitochondrial cristae shape determines the assembly and stability of respiratory chain supercomplexes and therefore the efficiency of mitochondrial respiration [11]. A major regulator of mitochondrial fusion and cristae structure is the optic atrophy-1 protein (OPA1) which resides in the inner mitochondrial membrane. OPA1 has a direct effect on metabolism by influencing the formation and stability of electron transport chain complexes within the cristae. Expression of OPA1 is decreased in both human and rat heart failure and decreased in mice [64] and cells subjected to hypoxia [10]. In cells and animal models, loss of OPA1 leads to abnormal cristae morphology, decreased supercomplex formation and reduced respiration efficiency [11]. Conversely, overexpression of OPA1 reduces infarct size after ischemic injury, increases mitochondrial fusion, cristae formation, respiration efficiency, supercomplex formation and ATP production (Fig. 7A) [10, 11, 59, 64]. On a molecular level, OPA1 protein can exist in a long form or can be proteolytically processed to a short form by two peptidases: YME1L and OMA1 [1]. The specific functions of long and short OPA1 are still under investigation; however, increased abundance of long OPA1 can increase mitochondrial fusion and protect from cardiac dysfunction [61]. Despite this post-transcriptional mode of regulation, there is currently no known epigenetic mechanism that regulates OPA1 expression. Interestingly, mice that overexpress OPA1 have a strikingly similar phenotype to our SMYD1a transgenic mice. Indeed, overexpression of SMYD1a resulted in larger mitochondria with more dense and narrower cristae, comparable to mice that overexpress OPA1. Therefore, we evaluated levels of OPA1 in our transgenic animals by western blotting. Our results show increased long OPA1 in mitochondria from TG mice (Fig. 7B–D) and, conversely, a decrease in both long and short OPA1 forms in *Smyd1-*KO mice (Fig. 7B–E). In addition, we also examined OPA1 expression in H9c2 cardiomyoblasts subjected to knockdown or overexpression of SMYD1a and observed that loss of *Smyd1* lead to down-regulation of OPA1, whereas SMYD1a overexpression increases OPA1 (Fig. 7F–I). Further, to investigate the mechanism by which SMYD1a controls OPA1 expression, we examined the *Opa1* loci using ChIP-qPCR in cultured H9c2 cardiomyoblasts (Fig. 7J–N). We observed significant enrichment of SMYD1a binding at the *Opa1* promoter (Fig. 7K), however, it did not correlate with enrichment of histone H3K4 trimethylation across the same region (Fig. 7L), suggesting SMYD1a regulates expression of this gene through a methyltransferase-independent mode, as has been seen with other methyltransferases (Supplementary Fig. S9) [54]. To affirm that SMYD1 regulates mitochondrial respiration through transcriptional control of OPA1, we analyzed mitochondrial respiratory capacity and conducted the Cell Mito Stress Test in H9c2 cardiomyoblasts. Consistent with previous findings [44] we observed decreased mitochondrial respiration in *Opa1*-KD cells, as reflected in lower basal, spare and maximal respiration and ATP production (Fig. 7O, P), Supplementary Fig. S10). As expected, overexpression of SMYD1a led to increased respiration capacity, but most importantly, overexpression of SMYD1a in the absence of *Opa1* did not rescue basal, maximal and spare respiration or ATP production (Fig. 7O, P). Overall, this data shows that SMYD1a regulates the expression of OPA1 in the heart.

## Discussion

In this report, we (1) demonstrate SMYD1a’s ability to positively regulate cardiac energetics in an animal model and protect from ischemic injury and (2) identify OPA1 as a novel downstream target of this myocyte-specific methyltransferase. To delineate how SMYD1a regulates energy efficiency and metabolism in the cardiomyocyte, we generated a novel mouse model capable of inducible cardiomyocyte-specific SMYD1a overexpression. When subjected to ischemic injury these transgenic mice display reduced infarct size and cardiomyocyte death concomitant with enhanced mitochondrial respiratory efficiency. In addition, our molecular analysis revealed that the cardiac tissue in these animals is protected from ischemic injury through SMYD1a’s synergistic regulation of two key mitochondrial pathways. First, SMYD1a maintains metabolic homeostasis by preserving the basal expression of PGC-1α and its downstream targets including electron transport chain subunits. Second, SMYD1a regulates the expression of OPA1, a key regulator of cristae morphology and ETC supercomplex formation by which it enhances mitochondrial respiration and ATP production.

**Fig. 8:**
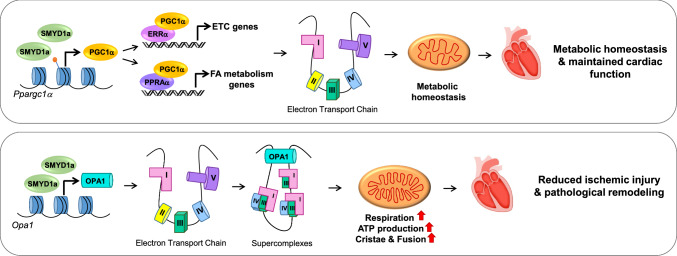
Schematic of SMYD1a’s role in the heart. Top panel: under basal conditions SMYD1a maintains metabolic homeostasis by regulating the expression of *Ppargc1a* through methylation of histone H3K4 at the promoter and its downstream targets including electron transport chain (ETC) subunits. Bottom panel: overexpression of SMYD1a regulates OPA1 expression which mediates cristae remodeling and supercomplex formation of respiratory chain complexes. This leads to enhanced mitochondrial respiration and ATP production to protect from ischemic injury and pathological remodeling in the heart

In the heart, it has been well established that PGC-1α regulates cardiac energetics and that its expression is decreased in the mouse heart after pressure overload-induced hypertrophy and in isolated cardiomyocytes treated with hypertrophic agonists [2]. This decrease in PGC-1α expression also corresponds to a decrease in PGC-1α target genes [38, 39]. In the failing human heart, PGC-1α expression has been shown to vary with most reports detecting a decrease and others showing no change [43]. This variability is likely due to the time points examined, therapeutic interventions administered and the presence of other comorbidities (including diabetes and obesity), not present in animal models. In addition, the expression of PGC-1α has also been shown to be critical for maintaining metabolic homeostasis where significant changes in expression (either increasing or decreasing PGC-1α expression) have been detrimental to cardiac function in mouse models [30, 31]. Therefore, maintaining basal levels of PGC-1α may be a key mechanism to maintain myocyte respiration and ATP production. While several transcription factors have been identified which can influence PGC-1α expression, the epigenetic mechanisms, which underlie transcriptional regulation of PGC-1α, are only beginning to be identified. Specifically, we previously identified SMYD1a as the only known epigenetic regulator of PGC-1α expression in the cardiomyocyte. In our new transgenic model presented here, we show that SMYD1a can regulate PGC-1α expression during ischemic injury. It’s interesting to note that increased SMYD1a does not increase PGC-1α expression beyond basal levels, which we believe is due to precisely controlled regulation (Fig. 8) and reflects the homeostatic balance between positive regulators of expression (like histone methyltransferases) and negative regulators (like histone demethylases). Indeed, previous reports have shown that an increase or decrease in PGC-1α in the heart leads to metabolic imbalances and cardiac dysfunction, highlighting the importance of maintaining normal levels [2, 5, 30, 31, 36, 46, 48]. However, while our data provide key molecular insights into SMYD1a’s regulation of PGC-1α and confirm that maintaining PGC-1α expression is necessary for metabolic homeostasis, it alone does not account for the increase in respiration capacity in our SMYD1a transgenic mice at physiological conditions.

Further evaluation of our TG mice demonstrated that the increased oxygen consumption is due to increased supercomplex formation of ETC complexes. These changes are influenced by increased mitochondrial cristae density, which is driven by OPA1, a master regulator of mitochondrial fusion and cristae morphology. In addition, we see reciprocal regulation of OPA1 expression in both our SMYD1a transgenic mice (where OPA1 is increased) and our *Smyd1* knockout mice (where OPA1 is decreased). Moreover, the phenotype in our SMYD1a gain-of-function mice is strikingly similar to the phenotype of transgenic mice which overexpress OPA1 where mild overexpression of OPA1 (1.6-fold increase) leads to increased cristae density, narrowing cristae junctions, increased supercomplex formation, enhanced respiration and protection from ischemic injury [59]. Overall, our results show OPA1 as a downstream target of SMYD1a and reveal a novel epigenetic pathway by which cardiomyocytes regulate cristae structure to dynamically adapt to energy demands. Our data clearly indicate that SMYD1a directly regulates OPA1 expression and demonstrates direct recruitment of SMYD1a to the *Opa1* promoter region. However, this binding does not correlate with the enrichment of the histone H3K4me3 at the same site but may imply regulation by a different mechanism (Supplementary Fig. S9). The canonical model regarding transcriptional regulation by methyltransferases suggests that each methyltransferase modifies a single, nonrandom, and specific amino acid residue on histones and influences transcription in only one direction: by either activating or repressing gene expression (Supplementary Fig. S9A). However, the most recent review of the literature suggests that the same methyltransferase can also modify different histone residue(s) to both activate or repress transcription (Supplementary Fig. S9B). In addition, some methyltransferases can also regulate gene expression through methylation of non-histone proteins, including transcription factors, chromatin remodeling proteins or other components of transcriptional machinery that regulate chromatin structure and its accessibility (Supplementary Fig. S9C). Finally, some methyltransferases have been shown to regulate transcription in a methyltransferase-independent mechanisms where they interact with (and do not methylate) other components of a chromatin-binding complex (Supplementary Fig. S9D). Therefore, we believe that the transcriptional regulation of OPA1 by SMYD1a could occur through either, (1) a methyltransferase-independent mechanism (which has been observed in other methyltransferases [54]) or, (2) by SMYD1a methylating a lysine residue other than K4 on histone H3 in the *Opa1* promoter, although to date no other lysine residues on histones have been shown to be methylated by SMYD1a. Ultimately, future studies will be needed to further interrogate the molecular mechanism driving this transcriptional regulation. In addition, the genome-wide profiling of genes regulated by SMYD1a’s histone methyltransferase activity (using ChIP-Seq methods) has never been carried out, instead only a few individual genes have been identified. Therefore, future work will be necessary to further characterize this pathway and identify other genes regulated by SMYD1a.

In conclusion, the data presented here provide a mechanistic basis for understanding how SMYD1a’s synergistic regulation of PGC-1α and OPA1 maintains the expression of electron transport chain subunits and remodels cristae structure, respectively, to enhance respiration and protect the heart from ischemic injury (Fig. 8). Ultimately, we highlight SMYD1a as an epigenetic regulator of mitochondrial respiration and provide broad implications for understanding how the cardiomyocyte upregulates energy efficiency to dynamically adapt to energy demands of the cell.

### Supplementary Information

Below is the link to the electronic supplementary material.

Supplementary file1 (PDF 1484 KB)

## Data Availability

All data generated or analyzed during this study are included in this published article and its supplementary information files.
