# Supplementary material for: SMYD1a protects the heart from ischemic injury by regulating OPA1-mediated cristae remodeling and supercomplex formation
Source: Basic Res Cardiol. 2023 May 22;118(1):20. doi: 10.1007/s00395-023-00991-6 (PMC10203008; doi:10.1007/s00395-023-00991-6)
Supplement: Supplementary file 1 — Supplementary file1 (PDF 1484 KB) [file 395_2023_991_MOESM1_ESM.pdf]

Supplementary Materials for

SMYD1a Protects the Heart from Ischemic Injury by Regulating OPA1 Mediated Cristae Remodeling and Supercomplex Formation.

Marta W. Szulik[1]*, Steven Valdez[1], Maureen Walsh[2], Kathryn Davis[1], Ryan Bia[1], Emilee Horiuchi[1], Sean O'Very[1], Anil K. Laxman[3], Linda Sandaklie-Nicolova[4], David R. Eberhardt[1], Jessica R. Durrant[5], Hanin Sheikh[1], Samuel Hickenlooper[1], Magnus Creed[1], Cameron Brady[1], Mickey Miller[1], Li Wang[1], June Garcia-Llana[1], Christopher Tracy[1], Stavros G. Drakos[1,6], Katsuhiko Funai[2], Dipayan Chaudhuri[1,6,7], Sihem Boudina[8], Sarah Franklin[1,6]*.

[1] Nora Eccles Harrison Cardiovascular Research and Training Institute, University of Utah, Salt Lake City, UT
[2] Diabetes & Metabolism Research Center, University of Utah, Salt Lake City, UT, USA
[3] Metabolic Phenotypic Core Facility, University of Utah, Salt Lake City, UT, USA
[4] Electron Microscopy Core Laboratory, University of Utah, Salt Lake City, UT, USA
[5] Dallas Tissue Research, Farmers Branch, TX, USA
[6] Division of Cardiovascular Medicine, Department of Internal Medicine, University of Utah, School of Medicine, Salt Lake City, UT, USA
[7] Department of Biochemistry, Department of Biomedical Engineering, University of Utah, Salt Lake City, UT, USA
[8] Department of Nutrition and Integrative Physiology, Program in Molecular Medicine, University of Utah, Salt Lake City, UT, USA.

*Denotes co-corresponding authors: Marta W. Szulik and Sarah Franklin.
Email: m.szulik@utah.edu, sarah.franklin@utah.edu

Table S1. Clinical characteristics of the study population. Left ventricular ejection fraction (LVEF) and left ventricular end diastolic diameter (LVEDD) at both LVAD implantation (pre-LVAD) and LVAD explantation/cardiac transplantation (post-LVAD) showing clinical differences between Responders and Non-Responders. (Values reported as Mean ± SEM).

**Variable**	**Responders N=5**	**Non-responders N=5**	***p*-value**
Male sex, (%)	4 (80)	4 (80)	
Age at LVAD implantation, years	56.2 ± 9.7	54.2 ± 7.6	0.876
**Pre-LVAD**			
LVEDD, cm	5.2 ± 0.5	6.2 ± 0.5	0.159
LVEF, %	38.8 ± 7.2	16.2 ± 3.1	0.017
**Post-LVAD**			
LVEDD, cm	5.0 ± 0.2	5.8 ± 0.6	0.277
LVEF, %	45.0 ± 3.9	23.6 ± 2.6	0.002
**HF Etiology**			
Ischemic cardiomyopathy, n (%)	2 (40.0)	2 (40.0)	
Non-ischemic cardiomyopathy, n (%)	3 (60.0)	3 (60.0)	
**New York Heart Association Functional Class**			
III, n (%)	2 (40.0)	2 (40.0)	
IV, n (%)	3 (60.0)	3 (60.0)	

Table S2. Clinical characteristics of the study population, donors. CVA – cardiovascular accident.

Variable	Donors, N=5, (Mean ± SEM)
**Female, n (%)**	5 (100%)
**Age**	38 ± 12
**Weight**	72 ± 16
**Cause of death**	
CVA Stroke	40%
Anoxia	40%
Head Trauma	20%

Table S3. List of primers used in this study.

β-Actin- mouse	Forward	TGT TAC CAA CTG GGA CGA
	Reverse	GGG GTG TTG AAG GTC TCA
Nppa (ANF)- mouse	Forward	CTG ATG GAT TTC AAG AAC CTG CT
	Reverse	CTC TGG GCT CCA ATC CTG TC
Myh6 (αMHC)- mouse	Forward	GAA CAG CTG GGA GAA GGG GG
	Reverse	GCC TCT GAG GCT ATT CIA TTG G
Myh7 (βMHC)- mouse	Forward	GAA CAG CTG GGA GAA GGG GG
	Reverse	GCC TCT GAG GCT ATT CTA TTG G
Apt2a2 (Serca2a)- mouse	Forward	CCT TCT ACC AGC TGA GTC ATT T
	Reverse	CAG ATG GAG CCC ACG ACC CA
Ppargc1 (PGC-1α)- mouse	Forward	CTT CGA GCT GTA CTT TTG TGG ACG GAA
	Reverse	CTC TGA GCT TCC TTC AGT AAA CTA TCA AA
Vim- mouse	Forward	TGA GAT CGC CAC CTA CAG GA
	Reverse	TGA TCA CCT GTC CAT CTC TGG
Col1a1- mouse	Forward	GGT CAG ACC TGT GTG TTC CC
	Reverse	GGT CCA TGT AGG CTA CGC TG
Smyd1a- mouse	Forward	GTG AAA TCC ATG TTT CAC ACG CAG ATG AG
	Reverse	CAG GAA GAG GTC GTC CTT CAG C
Smyd1b- mouse	Forward	CTG CAC TGT CAT ATT CAA CAA TGG CAA GAT
	Reverse	CAG GAA GAG GTC GTC CTT CAG C
Smyd1- human	Forward	GCC CAT TAC TGC GAC CGC AC
	Reverse	CTC CCC AAA GTG CTC CAC GTG G
Smyd1- mouse genotyping	Forward	CTC GCT CCG AGG GTT TGT ATC ACG
	Reverse	CTT ATC GTC GTC ATC CTT GTA ATC CAG G
Smyd2- mouse	Forward	GGA GGG CCA AAC ACT ACA AA
	Reverse	TGA GGG AGT ACA CGG GGT AG
Smyd3- mouse	Forward	TGA TGA AAG TTG GCA AGC TG
	Reverse	GTC CTT CTG GGG GTC CTT G
Smyd4- mouse	Forward	CGA ACC GGT AAA CCA GAG CA

	Reverse	ATG GCG AGT TTC AAC CAC CT
Smyd5- mouse	Forward	CTG ATT CGG AAG GGA GAG AC
	Reverse	TTC TGG TGG AGG TCC TTA CG
16S rRNA- mouse	Forward	CCG CAA GGG AAA GAT GAA AGA C
	Reverse	TCG TTT GGT TTC GGG GTT TC
ND1- mouse	Forward	CTA GCA GAA ACA AAC CGG GC
	Reverse	CCG GCT GCG TAT TCT ACG TT
Opa1- mouse/rat	Forward	ATA CTG GGA TCT GCT GTT GG
	Reverse	AAG TCA GGC ACA ATC CAC TT
Opa1 -1.0 kb	Forward	GGC GGG ACT TGT ATA GTG CT
	Reverse	CTC CCG GAA TCC TTA ATG GGG
Opa1 0.1 kb	Forward	CCC TCA GCA ACA AGG GCA TA
	Reverse	ACG TTA TCC GCG GCA CAT TT
Opa1 1.0 kb	Forward	TCG CAG ATG TTG TTG CCC TA
	Reverse	GTG CTA AGC TTG GGG GCT AT
Intergenic region (rat)	Forward	ATT TTG TGC TGC ATA ACC TCC T
	Reverse	TAG CAA CAT CCT AAG CTG GAC A
TBP - rat	Forward	GAA GTT TTG CAC CCC ATC CT
	Reverse	CAG GGG CCA ACT GAA AAC AG

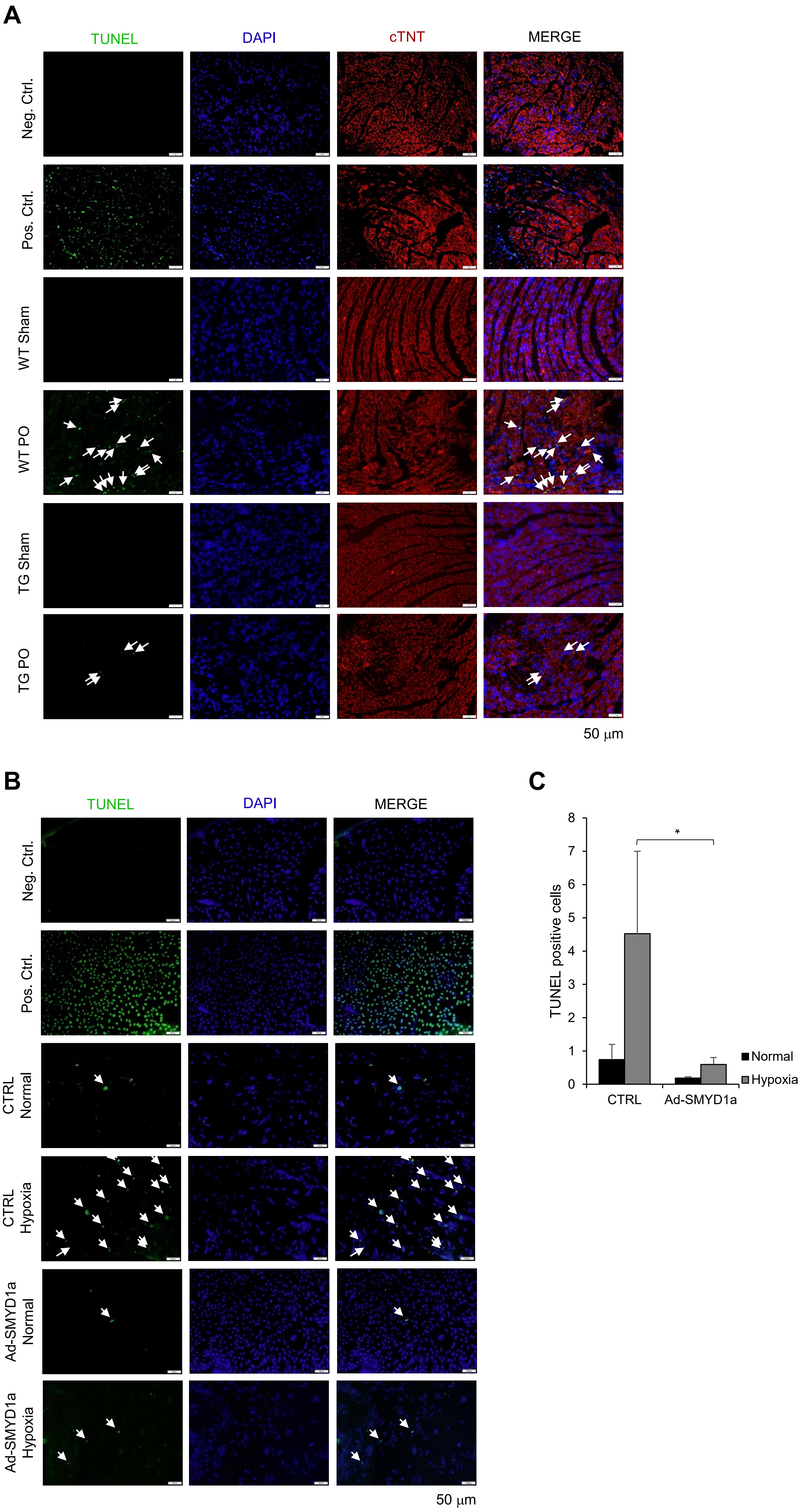

Fig. S1. **SMYD1a overexpression attenuates apoptotic cell death.** Apoptosis was detected by TUNEL assay in (**A**) cardiac tissue harvested 48h after PO (or Sham) surgery and in (**B**) H9c2 cardiomyoblasts that were overexpressing SMYD1a (by adenovirus) and subjected to hypoxic conditions. The results indicated significant decrease in apoptosis in TG PO mice when compared to WT PO group or in H9c2 cells overexpressing SMYD1a when compared to controls. DAPI staining shown in blue, cardiac troponin staining shown in red, TUNEL staining shown in GREEN and arrows point to TUNEL positive cells. Neg.Ctrl. indicates negative control and Pos.Ctrl. indicates positive control. Asterisk * indicates p<0.05, n=9-15.

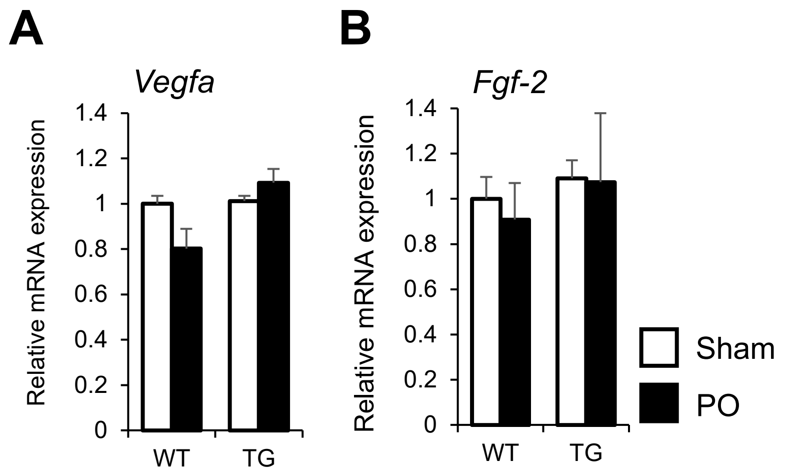

Fig. S2. **SMYD1a overexpression has no effect on angiogenesis in TG mice. A,B)** Markers of angiogenesis **A)** *Vegfa* and **B)** *Fgf-2* were measured by qRT-PCR and values expressed as relative mRNA intensities relative to WT control mice. n=4-6.

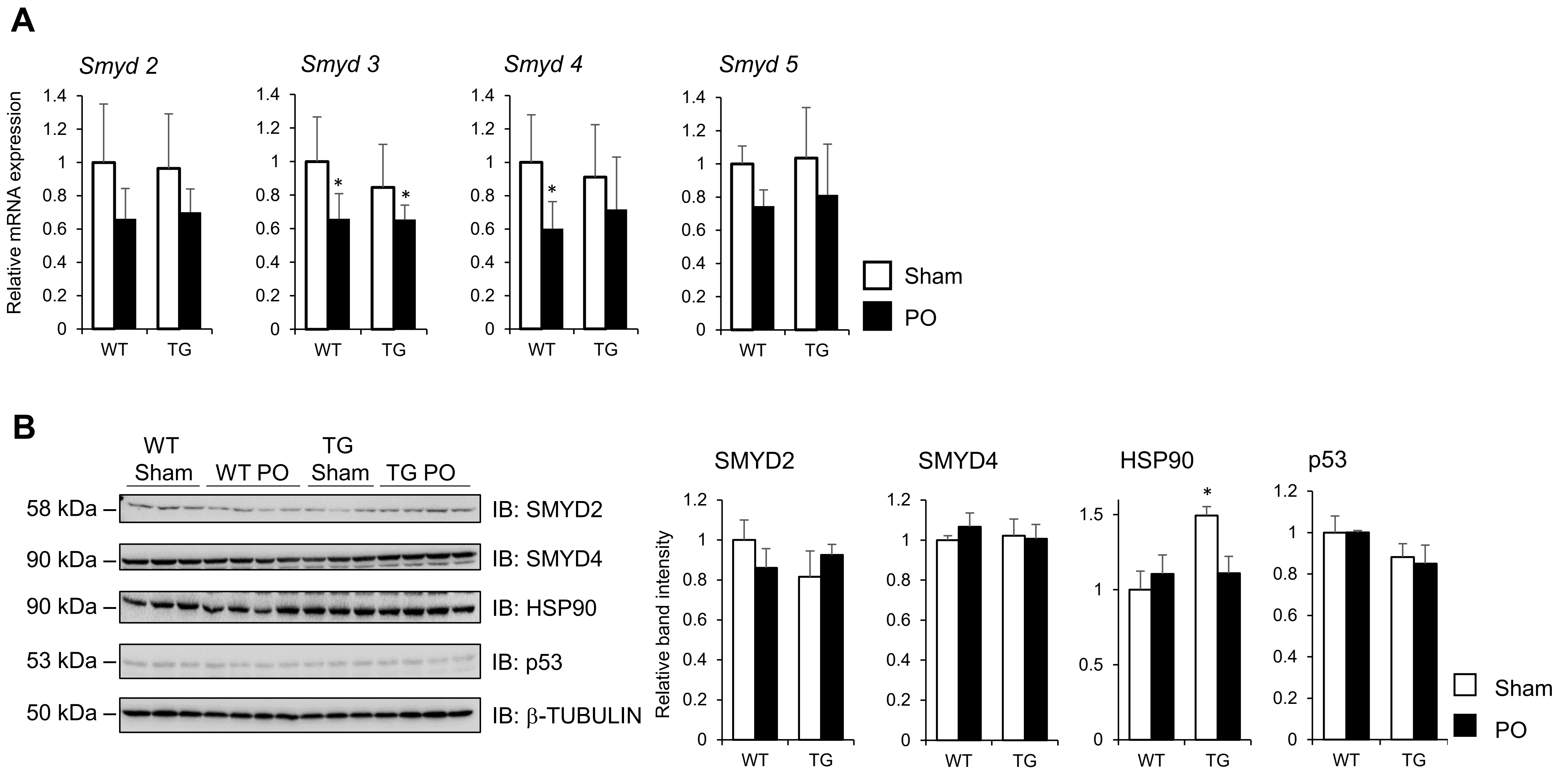

Fig. S3. **SMYD1a overexpression has no effect on expression of SMYD family members**. **A)** SMYD family members: *Smyd2, Smyd3, Smyd4* and *Smyd5* were measured by qRT-PCR and values expressed as relative mRNA intensities relative to WT control mice. Asterisk * indicates p<0.05 to sham control, n=4-6. **B)** As indicated by western blotting, overexpression of SMYD1a or permanent occlusion (PO) had no effect on levels of SMYD2, SMYD4 and p53, however there is a significant increase in HSP90 in TG Sham group as compared to WT controls. Asterisk * indicates p<0.05, n=3-4.

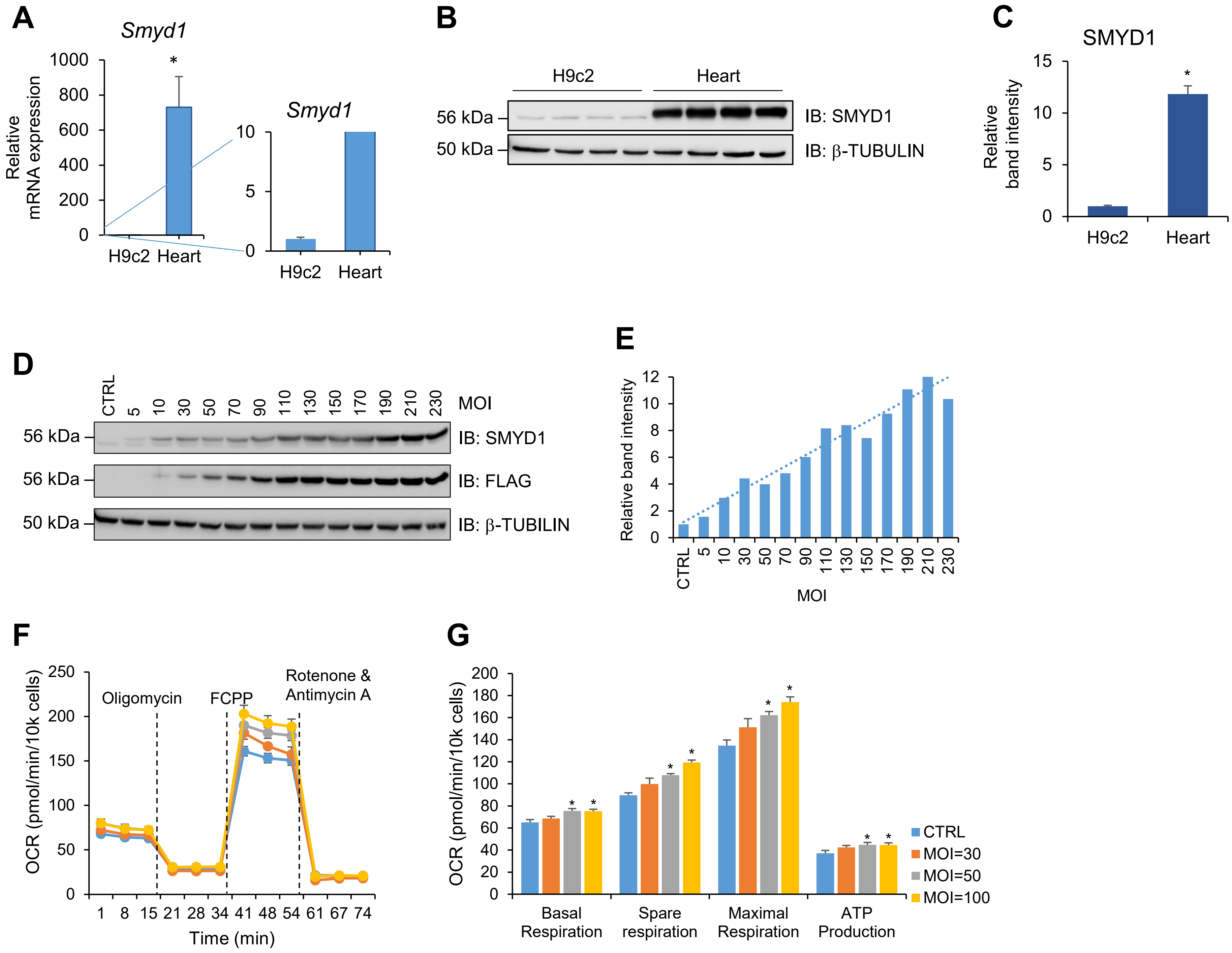

Fig. S4. **Basal expression of SMYD1a in cardiac tissue and H9c2 cardiomyoblasts.** Expression at both the transcript (**A**) and protein (**B,C**) levels shows that SMYD1's basal expression is much higher in cardiac tissue as compared to H9c2 cardiomyoblasts. **D,E**) Adenovirus mediated increasing overexpression of SMYD1a in H9c2 cells detected by western blotting and quantified. **F**) Cell Mito Stress test was conducted using a Seahorse Bioscience XFe96 analyzed by sequentially injecting 1mM oligomycin, 5mM FCCP, and 1mM rotenone+antymycin A inhibitors. H9c2 cardiomyoblasts were transduced with Ad-SMYD1a and oxygen consumption rates (OCR) were recorded and quantified in the presence of pyruvate as substrate. **G**) Quantitative analysis of mitochondrial OCR from H9c2 cells overexpressing SMYD1a indicates significant increases in respiration starting at MOI=50. Asterisk * indicates p<0.05, n=5-12.

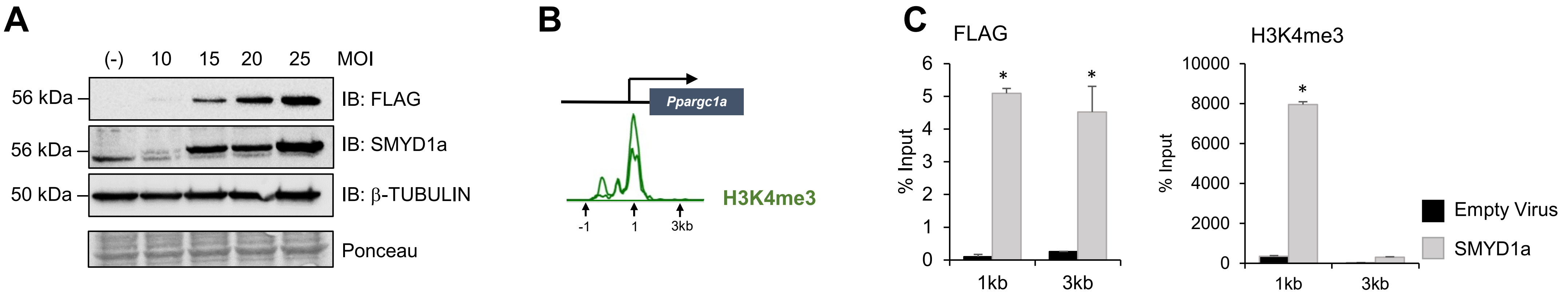

Fig. S5. **SMYD1a binds to the promoter of *Ppargc1α* and regulates its expression through histone H3K4me3 in isolated cells. A)** Adenoviral transduction of NRVMs led to a robust SMYD1a expression, as confirmed by western blotting. **B)** Publicly available ChIP-Seq data for histone H3K4me3 at the *Ppargc1α* promoter were used to design primers to use in ChIP-qPCR for SMYD1a-FLAG and histone H3K4me3 in isolated NRVMs showing **C)** binding at the *Ppargc1α* promoter, which increases trimethylation of histone H3K4me3 under basal conditions. Asterisk * indicates $p<0.05$, n=2.

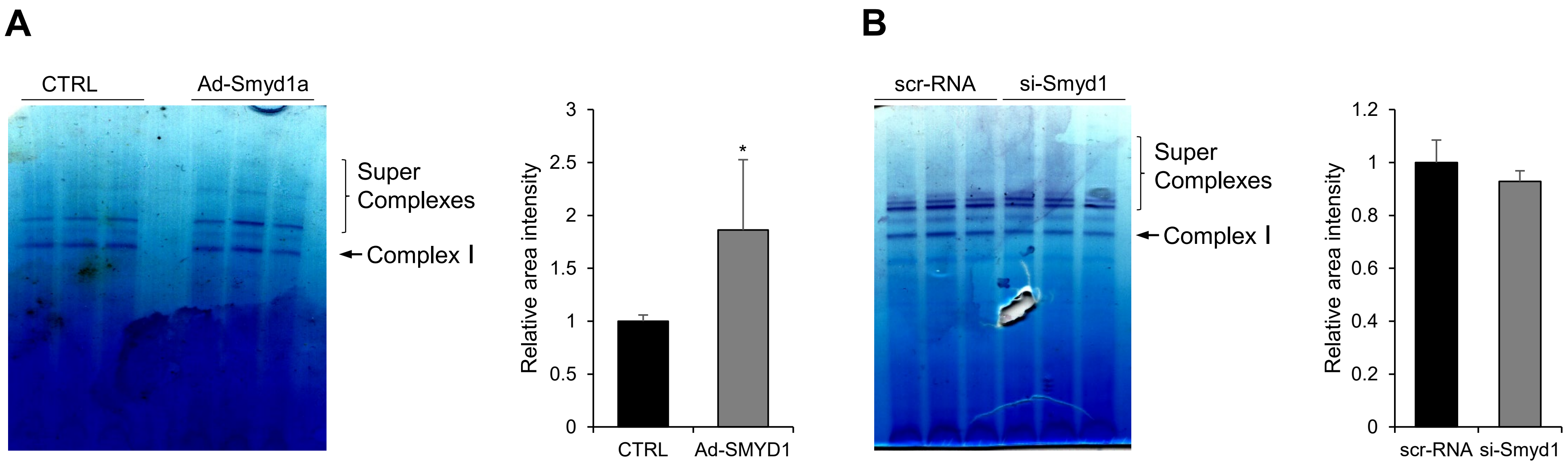

Fig. S6. **SMYD1a overexpression increases supercomplex formation in H9c2 cardiomyoblasts but knockdown of *Smyd1* has no effect.** Blue-native PAGE gel stained with Coomassie, evaluated for Complex I activity and quantified in H9c2 cardiomyoblasts 48h after A) adenovirus mediated overexpression of SMYD1a or B) si-RNA driven knockdown of *Smyd1*. Asterisk * indicates $p<0.05$, $n=3$.

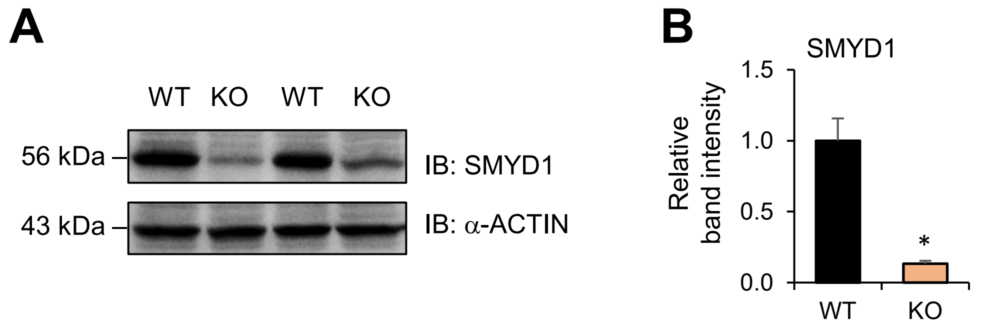

Fig. S7. **Cardiac specific deletion of *Smyd1*.** Mice fed tamoxifen diet for 3 weeks show nearly a complete loss of SMYD1 protein in the heart, as demonstrated by western blotting. WT – wild type, KO – *Smyd1* knockout. Asterisk * indicates $p<0.05$, n=3.

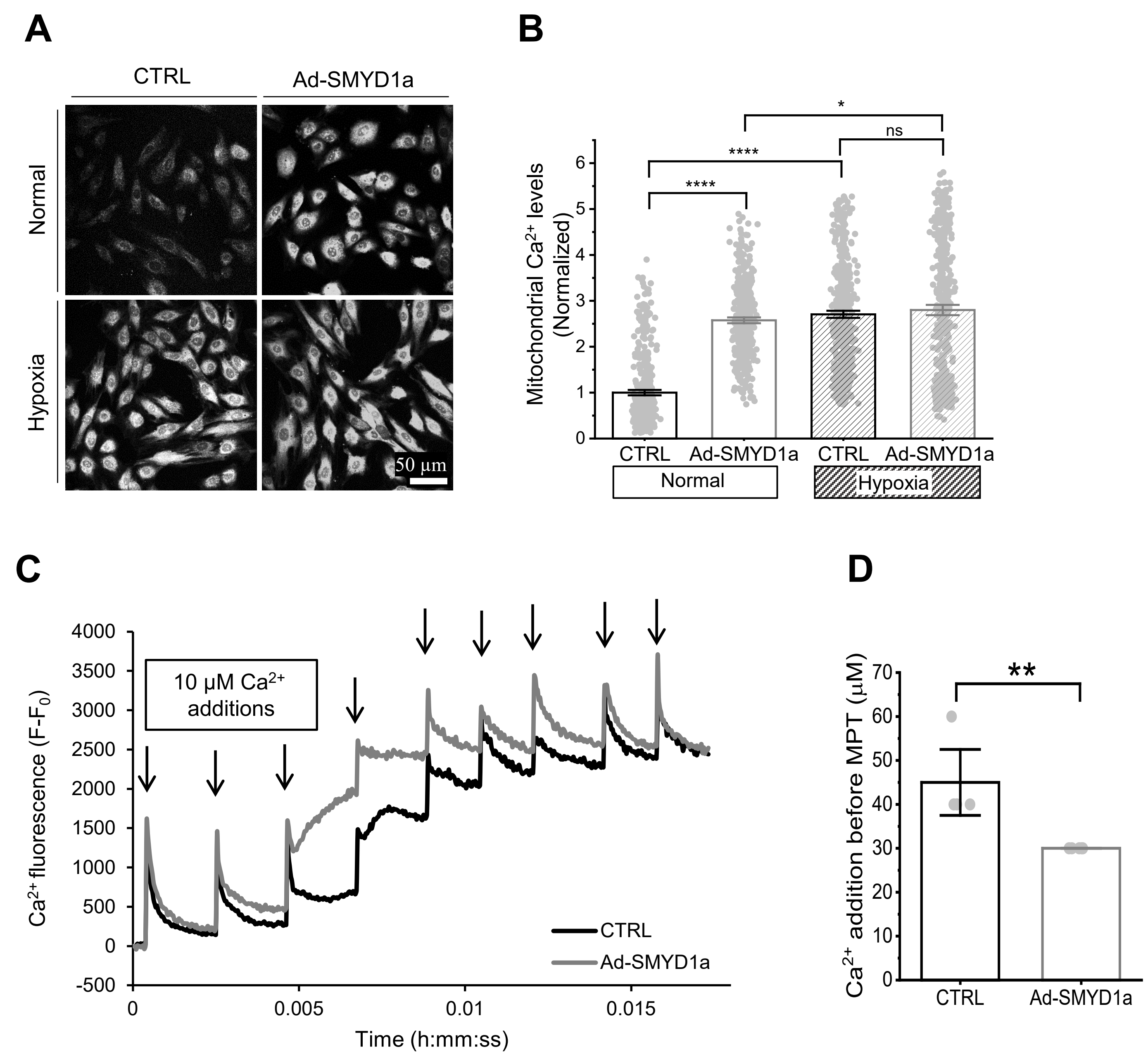

Fig. S8. SMYD1a overexpression induces mitochondrial calcium uptake but remains unchanged in hypoxic conditions. A) Representative images showing control and SMYD1a overexpressing H9c2 cardiomyoblasts stained with the mitochondrial Ca^{2+} sensor, XRhod1. **B)** Changes is mitochondrial Ca^{2+} levels following XRhod1 staining in control and SMYD1a overexpressing H9c2 cardiomyoblasts. Asterisk * indicates $p<0.05$, **** indicates $p<0.0001$, n=408-485. **C)** A representative calcium retention trace of control and SMYD1a overexpressing H9c2 cardiomyoblasts. Black arrows indicate 5mM Ca^{2+} injections. Mitochondrial viability was measured as ΔΨ/TMRM fluorescence. The graph is representative of five experiments each with 5E-5 cells. **D**) Comparison of calcium retention before the triggering of an MPT event in control and SMYD1a overexpressing cells. Asterisk ** indicates $p<0.01$, n=3.

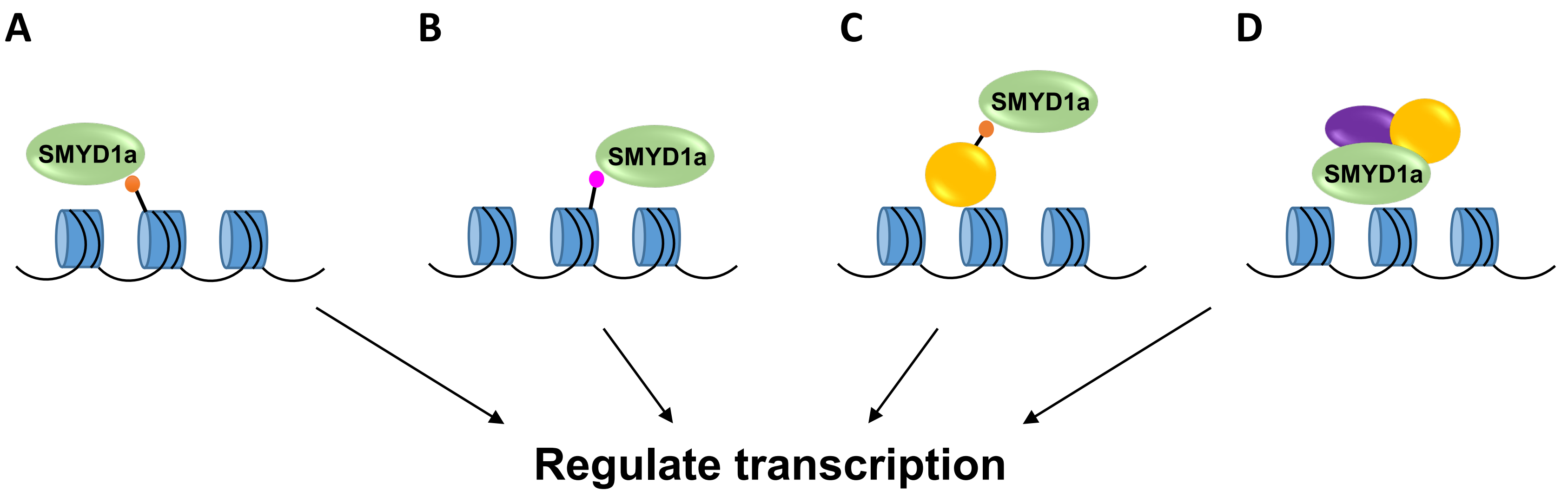

Fig. S9. **Schematic diagram representing possible mechanisms by which SMYD1a could regulate gene expression. A)** Canonically, SMYD1a activates transcription by trimethylation of histone H3 on lysine K4. **B)** However, based od data from other methyltransferases, it is possible that SMYD1a targets another histone PTM through which it regulates gene expression. **C)** In addition, SMYD1a could also regulate transcription by methylating other chromatin binding protein or **D)** through methyltransferase-independent mechanism, as a component of a chromatin binding complex. All of these four mechanisms have previously been shown to be utilized by methyltransferases to regulate gene expression.

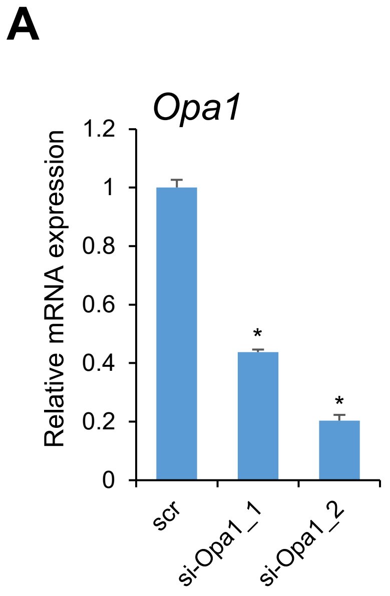

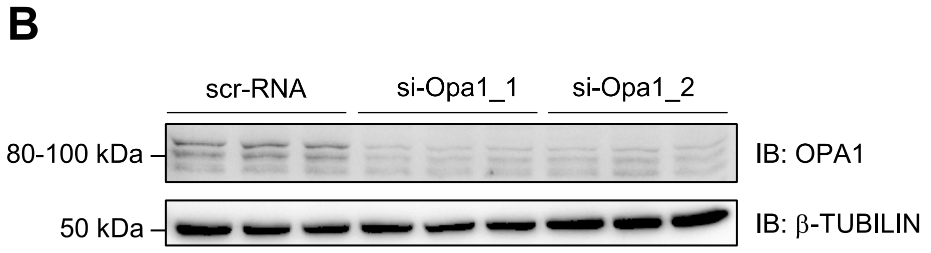

Fig. S10. **Knockdown of *Opa1*.** si-RNA-mediated knockdown of *Opa1* in H9c2 cardiomyoblasts showed significantly reduced levels of mRNA expression evaluated by (**A**) qRT-PCR and OPA1 protein as determined by (**B**) western blot analysis. Asterisk * indicates p<0.05, n=3.
